# Monocyte bioenergetics: An immunometabolic perspective in metabolic dysfunction-associated steatohepatitis

**DOI:** 10.1016/j.xcrm.2024.101564

**Published:** 2024-05-10

**Authors:** Moris Sangineto, Martina Ciarnelli, Tommaso Colangelo, Archana Moola, Vidyasagar Naik Bukke, Loren Duda, Rosanna Villani, Antonino Romano, Stefania Giandomenico, Hina Kanwal, Gaetano Serviddio

**Affiliations:** 1 C.U.R.E. (University Center for Liver Disease Research and Treatment), Liver Unit, Department of Medical and Surgical Sciences, University of Foggia, 71122 Foggia, Italy; 2 Department of Medical and Surgical Sciences, University of Foggia, 71122 Foggia, Italy; 3 Cancer Cell Signalling Unit, Fondazione IRCCS “Casa Sollievo della Sofferenza,” 71043 San Giovanni Rotondo (FG), Italy; 4 Pathology Unit, Department of Clinical and Experimental Medicine, University of Foggia, 71122 Foggia, Italy

**Keywords:** MASLD, MASH, immunometabolism, mitochondria, steatosis, monocyte, NASH, macrophage, dimethyl malonate, obesity

## Abstract

Monocytes (Mos) are crucial in the evolution of metabolic dysfunction-associated steatotic liver disease (MASLD) to metabolic dysfunction-associated steatohepatitis (MASH), and immunometabolism studies have recently suggested targeting leukocyte bioenergetics in inflammatory diseases. Here, we reveal a peculiar bioenergetic phenotype in circulating Mos of patients with MASH, characterized by high levels of glycolysis and mitochondrial (mt) respiration. The enhancement of mt respiratory chain activity, especially complex II (succinate dehydrogenase [SDH]), is unbalanced toward the production of reactive oxygen species (ROS) and is sustained at the transcriptional level with the involvement of the AMPK-mTOR-PGC-1α axis. The modulation of mt activity with dimethyl malonate (DMM), an SDH inhibitor, restores the metabolic profile and almost abrogates cytokine production. Analysis of a public single-cell RNA sequencing (scRNA-seq) dataset confirms that in murine models of MASH, liver Mo-derived macrophages exhibit an upregulation of mt and glycolytic energy pathways. Accordingly, the DMM injection in MASH mice contrasts Mo infiltration and macrophagic enrichment, suggesting immunometabolism as a potential target in MASH.

## Highlights

• Circulating monocytes (Mos) in patients with MASH show a bioenergetic reprogramming
• SDH inhibition *in vitro* restores MASH Mo bioenergetics, abolishing cytokine production
• In mice, energy pathways are upregulated in liver Mo-derived macrophages during MASH
• SDH inhibition *in vivo* reduces Mo infiltration and differentiation in MASH

## Graphical abstract

**Figure:**
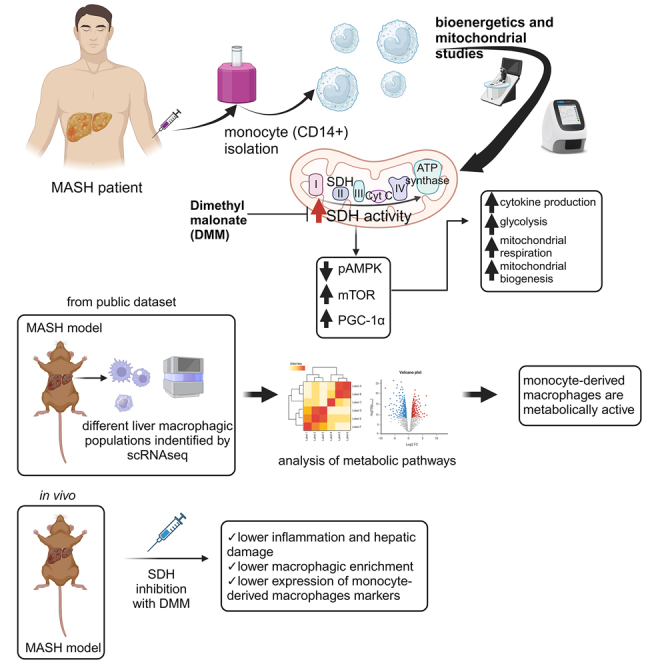

## Teaser

Sangineto et al. investigate the bioenergetics and mitochondrial activity of circulating monocytes in patients with MASH, revealing a hypermetabolic state also identified in liver monocyte-derived macrophages through transcriptomic analysis. Immunometabolic modulation via SDH inhibition attenuates inflammation both *in vitro* and *in vivo*, ameliorating MASH.

## Introduction

Metabolic dysfunction-associated steatohepatitis (MASH), the inflammatory subtype of metabolic dysfunction-associated steatotic liver disease (MASLD), represents a social and economic global burden with a growing incidence; it is predicted to become the first cause of liver transplantation in the United States by 2025, and its prevalence in European countries is expected to increase by more than 40% by 2030.^1^^,^^2^ MASH, previously known as non-alcoholic steatohepatitis, is characterized by steatosis, hepatocyte ballooning degeneration, and lobular inflammation, and it can further evolve to liver cirrhosis, a condition complicated by hepatocellular carcinoma.^3^^,^^4^ Although some studies reported that, according to the “multiple hit” hypothesis, several factors are involved in MASLD progression, such as insulin resistance, adipokines, high-fat (HF) diet, redox unbalance, and lipid metabolism dysregulation, the precise mechanisms that trigger the evolution of simple steatosis to MASH are still poorly understood.^5^ However, the direct involvement of innate immunity as a key factor is recently emerging.^6^ Kupffer cells (KCs), the resident liver macrophages (MØs), are activated by gut-derived pathogen-associated molecular patterns (PAMPs) (e.g., lipopolysaccharide [LPS]) or damage-associated molecular patterns (DAMPs), such as lipid peroxidation products generated by oxidative stress, via pattern recognition receptors. The consequence is the initiation of liver inflammation with the production of cytokines (e.g., interleukin [IL]-1β and tumor necrosis factor [TNF]-α), chemokines (e.g., CCL-2) and reactive oxygen species (ROS),^7^^,^^8^ leading to the recruitment of infiltrating monocytes (Mos), which generate a new resident population of MØs. These Mo-derived MØs (Mo-MØs) perpetuate and orchestrate inflammation in MASH.^9^^,^^10^ Recent studies of immunometabolism report that immune cells, including Mos, reprogram their bioenergetic phenotype to support inflammation. It is known that resting and regulatory phenotypes (e.g., M2 MØs or regulatory T cell lymphocytes) rely on oxidative phosphorylation, while immune “effectors'' (e.g., M1 MØs or Th17 lymphocytes) metabolically shift toward a glycolytic metabolism.^11^^,^^12^ The reason probably lies in the requirement of rapid ATP production and the synthesis of signaling molecules during the pro-inflammatory activity.^13^^,^^14^ However, the potential role of immunometabolism in chronic diseases is potently emerging,^15^ although it is very little explored in hepatic diseases.^16^ Therefore, considering the importance of Mo activity in determining MASLD progression and the urgent need to discover novel potential targets in MASH, we investigated the bioenergetic profile of circulating Mos in patients with MASH.

## Results

### MASH Mos show a metabolic reprogramming

**Figure 1:**
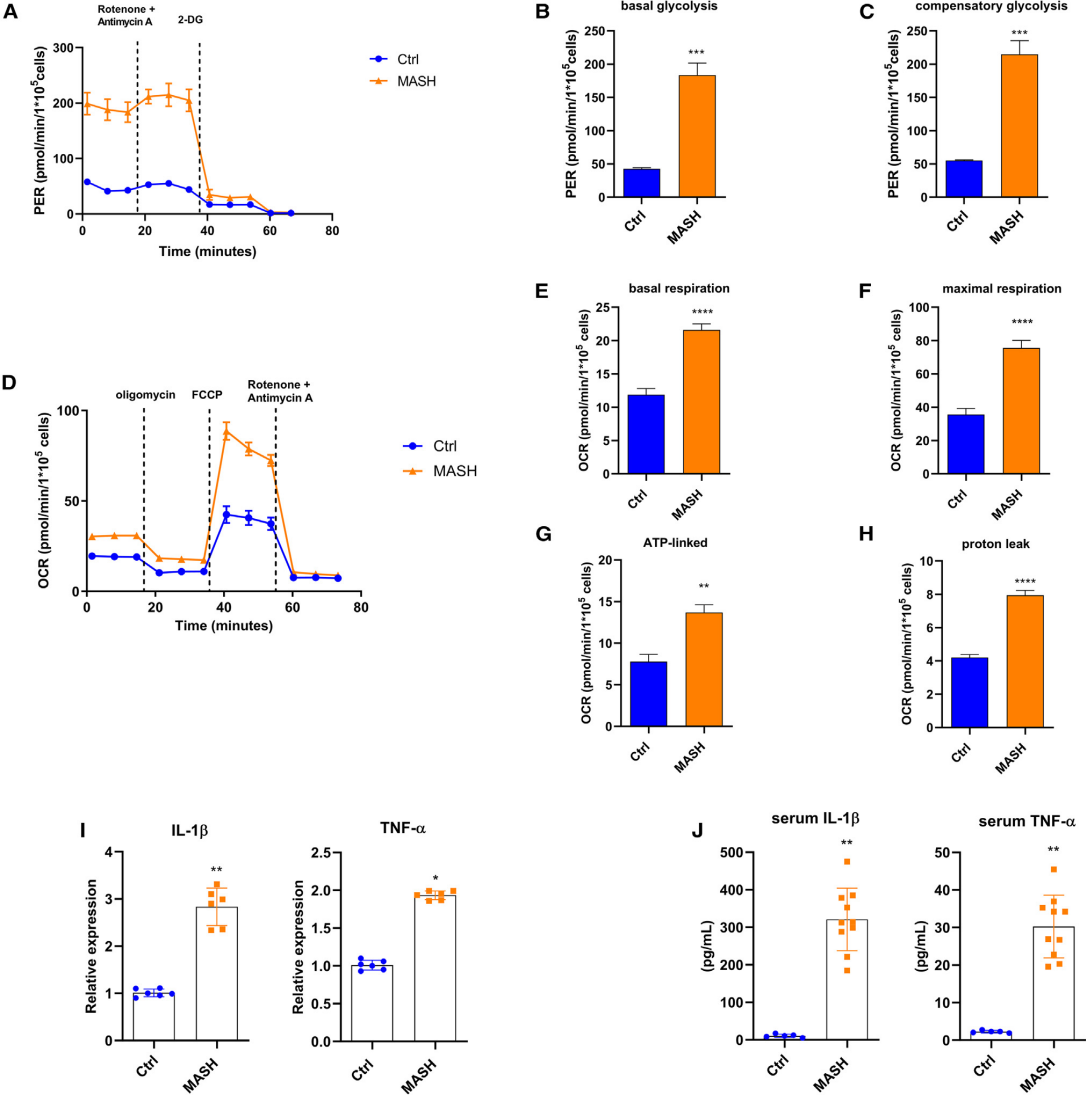
MASH Mos show metabolic reprogramming (A–H) Glycolysis determined by measuring PER (A–C) and mitochondrial respiration determined by measuring OCR (D–H) in healthy control (Ctrl) and MASH Mos (*n* = Ctrl: 5, MASH: 8; each subject analyzed in duplicate). (I) Relative mRNA expression of pro-inflammatory cytokines (*Il-1β* and *Tnf-α*) in Ctrl and MASH Mos (*n* = 6 per group) determined by qPCR. (J) IL-1β and TNF-α protein levels in serum of Ctrls and patients with MASH determined by ELISA (*n* = Ctrl: 5, MASH: 10). Data are expressed in mean ± SEM; ∗*p* < 0.05, ∗∗*p* < 0.01, ∗∗∗*p* < 0.001, and ∗∗∗∗*p* < 0.0001 according to two-tailed Student’s t test. Mo, monocyte; PER, proton efflux rate; OCR, oxygen consumption rate.

Considering the importance of Mos in the inflammatory orchestration in MASH, we dissected the bioenergetic profile of MASH patient Mos *ex vivo* by using a Seahorse XF HS Mini Analyzer (Agilent Technologies). The “glycolysis rate assay” (Agilent Technologies) permits calculating glycolysis with extreme precision, subtracting the mitochondrial-derived acidification, and revealing rapid metabolic switches not detectable in lactate assays, hence making it a valuable method to give information about bioenergetic phenotypes. Interestingly, glycolysis, measured by proton efflux rate, was significantly higher in MASH Mos compared to healthy control (Ctrl) Mos (Figures 1A and 1B). The injection of rotenone and antimycin A, inhibitors of mitochondrial complexes I and III, respectively, shows the capability of cells to augment glycolysis in response to mitochondrial ATP deprivation, a process known as compensatory glycolysis. MASH Mos displayed a considerable increase of compensatory glycolysis, highlighting a propensity to supply a large amount of energy by glycolysis (Figure 1C). Mitochondrial respiration was determined by the oxygen consumption rate (OCR) at the basal state and after inhibitor injection (i.e., oligomycin, carbonyl cyanide-p-trifluoromethoxyphenylhydrazone [FCCP], and rotenone/antimycin A). Interestingly, MASH Mos displayed a significant increase of basal respiration (Figures 1D and 1E). Moreover, the block of ATP synthase with oligomycin permitted us to estimate the quote of respiration devoted to ATP production, highlighting that the mitochondrial respiration gain observed in MASH Mos was not completely finalized to ATP synthesis and instead generated a considerable proton leak (Figures 1D–1H). The injection of the uncoupler FCCP can dissipate the proton gradient and maximize the OCR, assessing the maximum capacity that the electron transport chain (ETC) can achieve, namely the maximal respiration. Interestingly, MASH Mos reached very high levels of maximal respiration (Figures 1D and 1F). Overall, these data underline that MASH Mos present a hypermetabolic state characterized by a considerable increase of glycolysis and mitochondrial respiration enhancement associated with proton leak. This bioenergetic phenotype was linked to a pro-inflammatory activity, as shown by the higher expression of *Il-1β* and *Tnf-α* (Figure 1I), which was in line with serum levels (Figure 1J).

**Figure 2:**
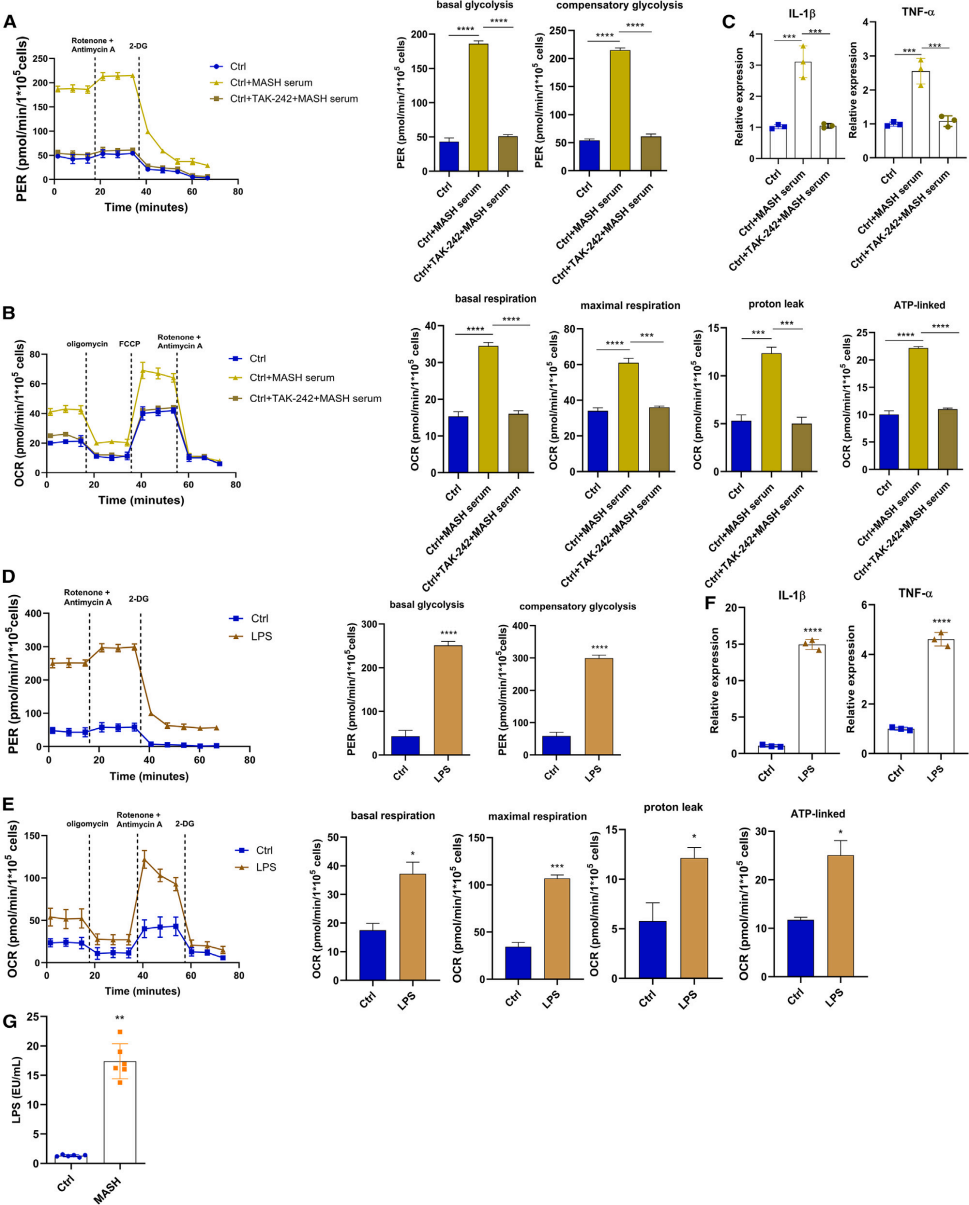
Metabolic reprogramming in Mos is induced by TLR4 (A–C) Glycolysis determined by measuring PER (A), mitochondrial respiration determined by measuring OCR (B), and relative expression of *Il-1β* and *Tnf-α* determined by qPCR (C) in Mos of Ctrls untreated or stimulated with 10% MASH serum for 4 h ± pre-treatment with the TLR4 inhibitor TAK-242 (1 μM) for 1 h (3 experiments performed in duplicate). (D–F) Glycolysis determined by measuring PER (D), (E) mitochondrial respiration determined by measuring OCR (E), and relative expression of *Il-1β* and *Tnf-α* determined by qPCR (F) in Mos of Ctrls untreated or stimulated with LPS (50 ng/mL) for 4 h (3 experiments performed in duplicate). (G) Quantification of endotoxin (LPS) concentration in serum of Ctrls and patients with MASH determined by using the Pierce Chromogenic Endotoxin Quant Kit (Thermo Fisher Scientific) (*n* = 6 per group). Data are expressed in mean ± SEM; ∗*p* < 0.05, ∗∗*p* < 0.01, ∗∗∗*p* < 0.001, and ∗∗∗∗*p* < 0.0001 according to two-tailed Student’s t test or one-Way ANOVA followed by post hoc analysis (Bonferroni test). LPS, lipopolysaccharide; Mo, monocyte; PER, proton efflux rate; OCR, oxygen consumption rate.

In order to define whether a MASH serum mediator could affect the Mo metabolic profile, we exposed Ctrl Mos to the serum of patients with MASH and obtained a bioenergetic profile very similar to MASH Mos in terms of both glycolysis and mitochondrial respiration (Figures 2A and 2B). Since it is known that patients with MASH show intestinal barrier defects, hence hypothesizing a role of PAMPs in the MASH Mo bioenergetic profile, we treated Ctrl Mos with MASH serum and TAK-242, a selective inhibitor of Toll-like receptor 4 (TLR4). Very interestingly, the TLR4 inhibition abolished the bioenergetic repurposing and cytokine expression induced by patients’ sera (Figures 2A–2C). In order to corroborate this observation, we stimulated Ctrl Mos with LPS, observing again the induction of the hypermetabolic state characterized by an increase of glycolysis and mitochondrial respiration with proton leak (Figures 2D–2F). Along these lines, we might speculate that TLR4 activation, probably due to circulating LPS and/or other PAMPs/DAMPs, plays a pivotal role in Mo pro-inflammatory activity and the related bioenergetic reprogramming observed in patients with MASH. Of note, circulating LPS levels were much higher in subjects with MASH (Figure 2G).

### Mitochondrial dysfunction in MASH Mos

**Figure 3:**
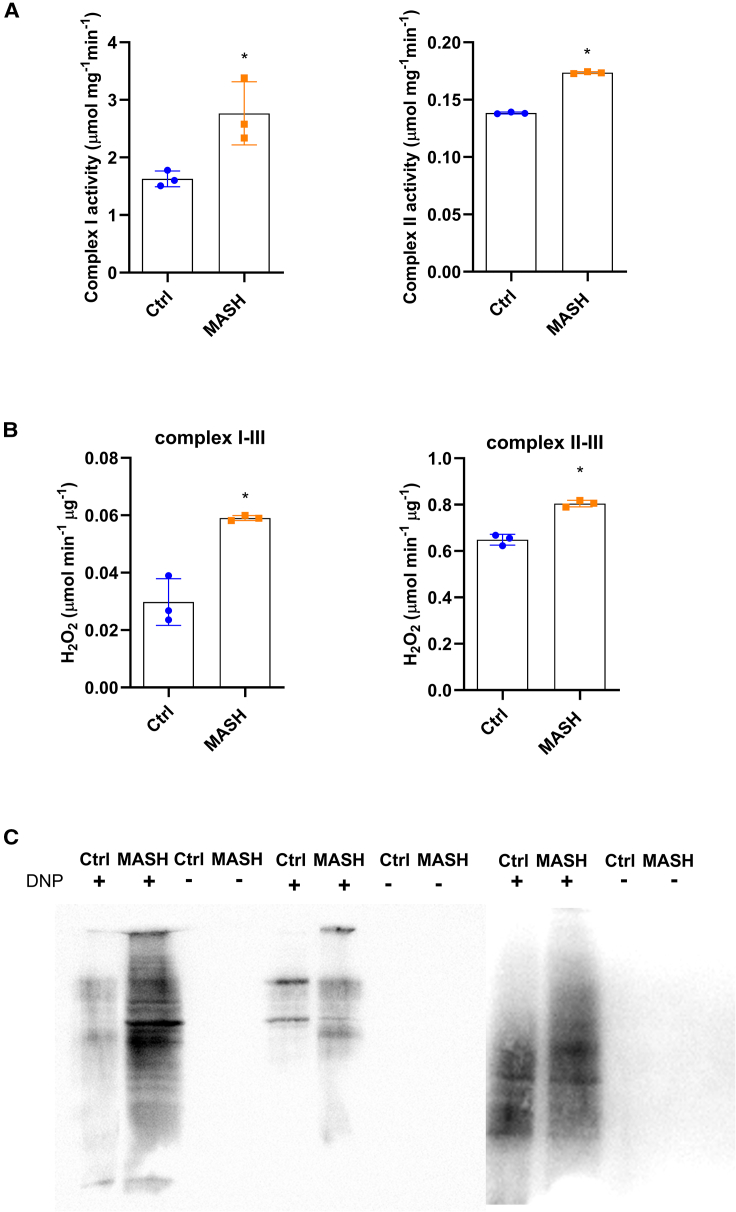
Mitochondrial dysfunction in MASH Mos (A) Respiratory chain complex I and II enzymatic activity determined spectrophotometrically in Ctrl and MASH Mos (*n* = 3 per group). (B) Peroxide production from pyruvate/malate (complex I–III activity) and succinate (complex II–III activity) in Ctrl and MASH Mos (*n* = 3 per group). (C) Pictures of mitochondrial oxidized proteins detected with Oxyblot (Millipore Bioscience Research Reagents) in Ctrl and MASH Mos (*n* = 3 per group). Data are expressed in mean ± SEM; ∗*p* < 0.05 according to two-tailed Student’s t test. Mo, monocyte.

**Figure 4:**
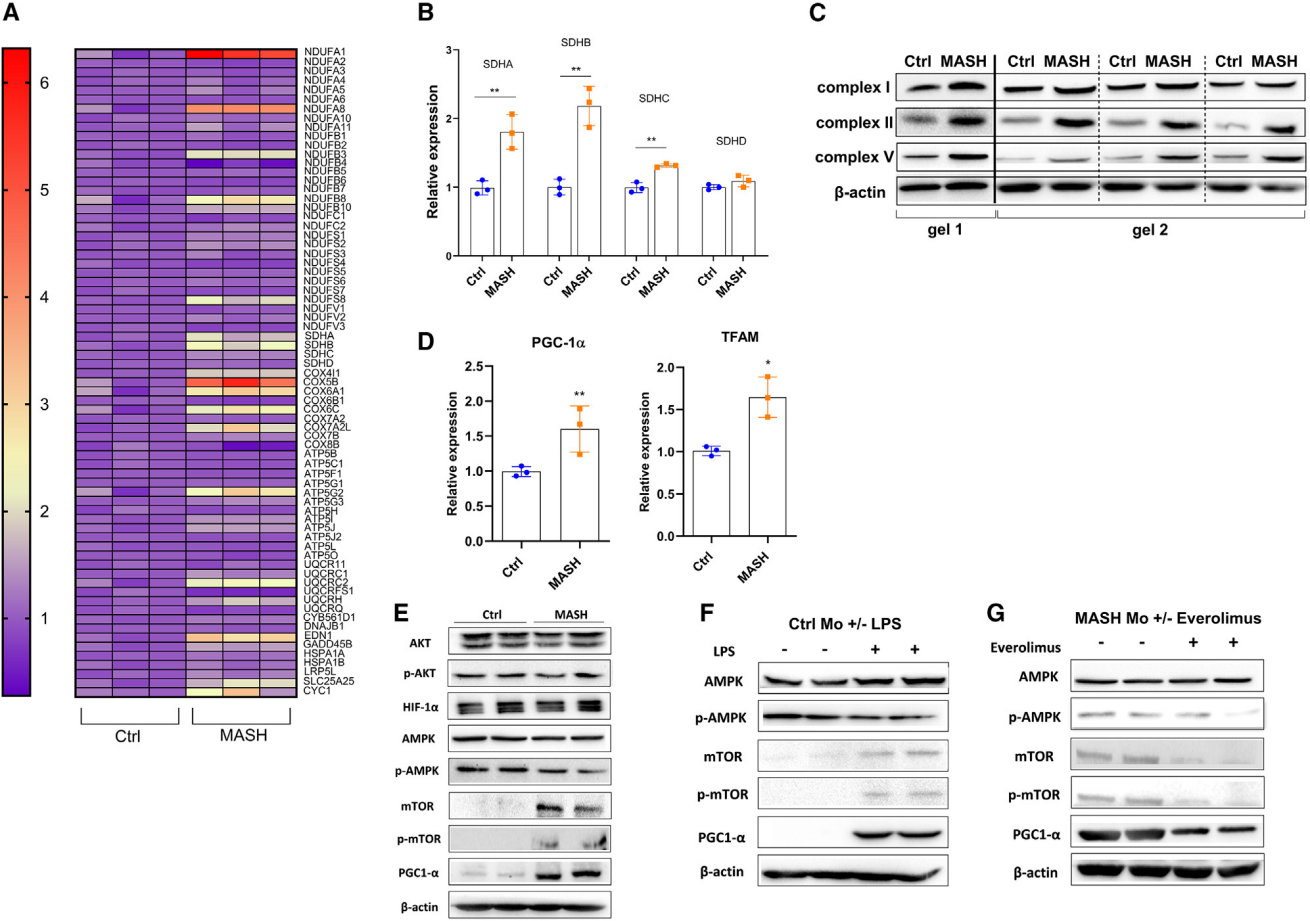
Mitochondrial activity is sustained at the transcriptional level in MASH Mo (A) Heatmap plot of Mo differential expressed genes involved in mitochondrial energy metabolism from the comparison between Ctrl and MASH patients (n= 3 per group); high expression is indicated in red and low expression is indicated in violet. (B) Relative mRNA expression of *Sdh* subunits in Ctrl and MASH Mo (n= 3 per group). A and B determined by qPCR using PrimePCR™ array “Mitochondria Energy Metabolism Plus” (Bio-Rad Laboratories Inc). (C) Pictures of protein levels of respiratory chain complexes I, II and V and β-actin as loading control, in Ctrl and MASH Mo determined by Western blot analysis (n= 4 per group; 1 MASH patient vs 1 ctrl performed on gel 1; 3 MASH patients vs 3 ctrls performed on gel 2). (D) Relative mRNA expression of *Pgc-1α* and *Tfam* in Ctrl and MASH Mo determined by qPCR (n= 3 per group). (E) Pictures of protein levels of AKT, p-AKT, HIF-1α, AMPK, p-AMPK, mTOR, p-mTOR, PGC-1α, and β-actin as loading control, in Ctrl and MASH patients Mo, determined by Western blot analysis (n=2 per group). (F) Pictures of protein levels of AMPK, p-AMPK, mTOR, p-mTOR, PGC-1α, and β-actin as loading control, in Ctrl Mo untreated or treated with LPS (50 ng/mL) for 4 h, determined by Western blot analysis (n= 2 per group). (G) Pictures of protein levels of AMPK, p-AMPK, mTOR, p-mTOR, PGC-1α, and β-actin as loading control, in MASH Mo untreated or treated with mTOR inhibitor, everolimus (10 nM) for 4 h, determined by Western blot analysis (n= 2 per group). Data are expressed in mean ± SEM; ^∗^p<0.05, according to two-tails student’s t-test. SDH, Succinate dehydrogenase; AKT, Protein kinase B; p-AKT, phosphor-protein kinase B; HIF-1α, Hypoxia-inducible factor 1-α; PGC-1α, Peroxisome proliferator-activated receptor-gamma coactivator-1α; TFAM, Transcription Factor A, Mitochondrial; AMPK, AMP-activated protein kinase; p-AMPK, phospho-AMP-activated protein kinase; mTOR, mechanistic target of rapamycin kinase; phospho-mTOR, mechanistic target of rapamycin kinase; LPS, lipopolysaccharide; Mo, Monocytes.

In a second step, we decided to explore the mitochondrial function. In line with the increase of basal and maximal mitochondrial respiration, the spectrophotometric analysis revealed that MASH Mos were characterized by the enhancement of ETC complex I and complex II enzymatic activity (Figure 3A). However, the mitochondrial respiratory chain is also the major source of ROS under pathological conditions,^17^ and here, we have shown a considerable proton leak in MASH Mos. Therefore, quantifying the H_2_O_2_ production rate by using pyruvate/malate (for complex I) and succinate (for complex II) as mitochondrial substrates, we found that MASH Mos showed high levels of peroxide production from both complex I and complex II activities (Figure 3B). Moreover, the Oxyblot analysis (Millipore Bioscience Research Reagents) revealed that the total quantity of oxidized proteins in mitochondria was significantly higher in the Mos of subjects with MASH (Figure 3C). Overall, these data suggested that MASH Mos presented mitochondrial dysfunction with ROS production and consequent oxidative stress. The expression studies conducted by using the PrimePCR array Mitochondria Energy Metabolism Plus (Bio-Rad Laboratories) showed that out of 78 targets analyzed (Table S1), 39 genes were significantly dysregulated. In particular, only 7 genes were downregulated, while 32 genes were upregulated, in MASH Mos compared to Ctrl Mos (Figures 4A and S1). Most of these genes encode for ETC subunits such as *NADH:ubiquinone oxidoreductase* (*Nduf*), *Ubiquinol-cytochrome c reductase* (*Uqcr*), *Cytochrome c oxidase* (*Cox*), and *ATP synthase* (*Atp5*) (Figure S1; Table S1), and in particular, 3 out of 4 *Succinate dehydrogenase* (*Sdh*) subunits were more expressed in MASH Mos as well (Figure 4B). In accordance with this, the protein levels of complex I, II, and V subunits were more represented in MASH Mos (Figure 4C). It was interesting to note that *Transcription factor A, mitochondrial* (*Tfam*), a key activator of the mitochondrial genome transcription, and *Peroxisome-proliferator-activated receptor-gamma coactivator-1α* (*Pgc1-α*), a master regulator of the mitochondrial biogenesis,^18^^,^^19^ were both significantly upregulated in patient Mos (Figure 4D). It is conceivable to believe that in MASH Mos, the transcription and production of ETC subunits support the requirement to increase mitochondrial respiration, which is in turn mostly addressed to ROS production. Very importantly, by analyzing potential mediators of metabolic adaptations in pro-inflammatory leukocytes, we found a significant increase of mammalian target of rapamycin (mTOR) (total and phosphorylated) and PGC-1α with a reduction of 5' AMP-activated protein kinase (AMPK) phosphorylation (Figure 4E). On the contrary, protein kinase B (Akt) phosphorylation and hypoxia-inducible factor 1 alpha (HIF-1α) were not different between Ctrl and MASH Mos (Figure 4E). This is important, as lower AMPK phosphorylation favors mTOR activity, which is known to be involved in glycolysis induction during macrophagic M1 polarization.^20^ Moreover, mTOR has been associated with PGC-1α activation.^21^ Interestingly, a similar profile (i.e., low phospho-AMPK [p-AMPK], high mTOR, and PGC-1α) with ETC subunit transcription was exhibited by Ctrl Mos when exposed to LPS (Figures 4F and S2), suggesting again a role of TLR4 activation, while the exposure of MASH Mos to everolimus, an mTOR-selective inhibitor, restored mTOR and PGC-1α levels, with no improvement in AMPK phosphorylation, confirming a link between mTOR and PGC-1α (Figure 4G). Collectively, these findings suggest that in MASH Mo metabolism, the AMPK-mTOR-PGC-1α axis is primarily involved.

### DMM modulates MASH Mo bioenergetics and reduces cytokine production

**Figure 5:**
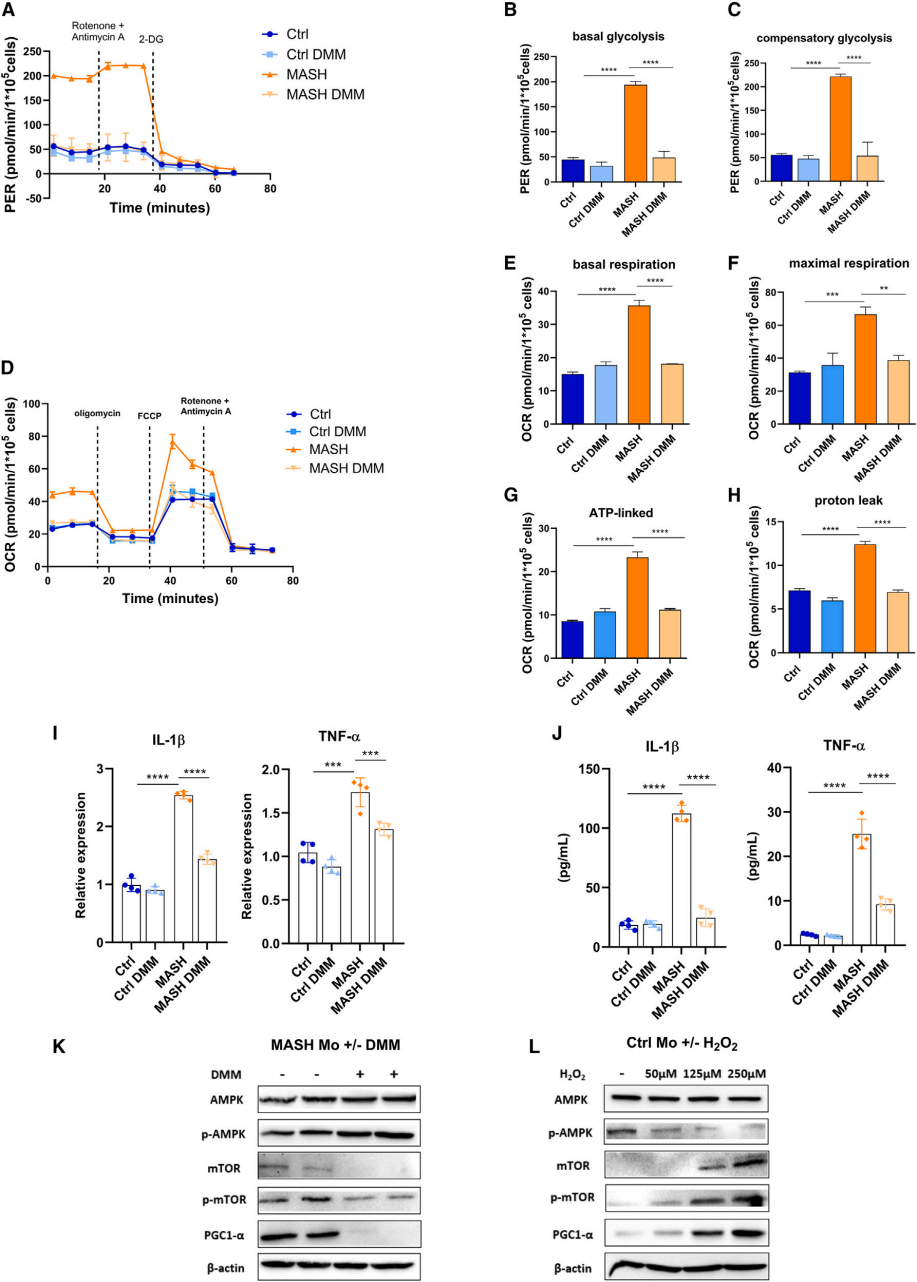
SDH inhibition effect on MASH Mo bioenergetic and cytokine production (A–H) Glycolysis determined by measuring PER (A–C) and mitochondrial respiration determined by measuring OCR (D–H) in Ctrl and MASH Mos ± DMM (10 mM) for 4 h (experiments performed by 3 Ctrls and 3 patients, each subject studied in duplicate). (I) Relative mRNA expression of pro-inflammatory cytokines (IL-1β and TNF-α) in Ctrl and MASH Mos ± DMM (10 mM) for 4 h (*n* = 4 experiments performed in duplicate) determined by qPCR. (J) IL-1β and TNF-α protein levels in Mo supernatants of Ctrls and MASH patients ± DMM (10 mM) for 4 h determined by ELISA (*n* = 4 per group). (K) Pictures of protein levels of AMPK, p-AMPK, mTOR, p-mTOR, and PGC-1α and β-actin as loading control in MASH Mos untreated or treated with DMM (10 mM) for 4 h (*n* = 2 per group). (L) Pictures of protein levels of AMPK, p-AMPK, mTOR, p-mTOR, and PGC-1α and β-actin as loading control in Ctrl Mos untreated or treated with increasing concentrations of H_2_O_2_ (50, 125, and 250 μM) for 4 h. Data are expressed in mean ± SEM; ∗*p* < 0.05, ∗∗*p* < 0.01, ∗∗∗*p* < 0.001, and ∗∗∗∗*p* < 0.0001 according to one-way ANOVA followed by post hoc analysis (Bonferroni test). DMM, dimethyl malonate; IL-1β, interleukin-1β; TNF-α, tumor necrosis factor-α; Mo, monocyte; AMPK, AMP-activated protein kinase; p-AMPK, phospho-AMP-activated protein kinase; mTOR, mechanistic target of rapamycin kinase; phospho-mTOR, mechanistic target of rapamycin kinase; PGC-1α, peroxisome proliferator-activated receptor-gamma coactivator-1α; PER, proton efflux rate; OCR, oxygen consumption rate..

Considering the immunometabolic alterations observed in MASH Mos, we questioned whether targeting the cellular bioenergetic, to control Mo inflammatory activity, might be a potential strategy for MASH. To do this, Mos have been treated with dimethyl malonate (DMM), a molecule that inhibits SDH activity. In fact, Mills et al. recently demonstrated that the activity of SDH, a key enzyme in the Krebs cycle and in the ETC (as complex II), is crucial in promoting metabolic reprogramming and pro-inflammatory activity in MØs,^22^ and SDH inhibition with DMM has been reported to exert important immunomodulatory effects.^23^^,^^24^^,^^25^ Moreover, we have shown that in MASH Mos, SDH is highly expressed, and its activity is augmented and unbalanced toward hydrogen peroxide production. Therefore, we examined the bioenergetic profile of MASH Mos exposed to DMM, which significantly dampened glycolysis and compensatory glycolysis (Figures 5A–5C). Moreover, as shown by OCR levels (Figure 5D), DMM-treated Mos showed normal levels of basal and maximal respiration (Figures 5E and 5F), with a consequent control of proton leak (Figures 5G and 5H), compared to untreated Mos. As expected, the production of the pro-inflammatory cytokines IL-1β and TNF-α was reduced, as demonstrated by qPCR (Figure 5I) and ELISA (Figure 5J). Interestingly, DMM increased p-AMPK levels and reduced mTOR and PGC-1α, underlining again the role of the AMPK-mTOR-PGC-1α pathway in the metabolic and pro-inflammatory activity in MASH Mos (Figure 5K). Accordingly, the expression of most dysregulated ETC subunits and *Tfam* was restored by DMM (Figures S3A and S3B). Considering the demonstrated ability of DMM to modulate bioenergetic and pro-inflammatory function in MØs by reducing ROS production,^22^^,^^23^ we have exposed healthy Mos to hydrogen peroxide. Interestingly, we observed a dose-dependent induction of mTOR and PGC1-α protein levels, with AMPK dephosphorylation, by increasing the amount of H_2_O_2_ (50, 125, and 250 μM) (Figure 5L), confirming the role of oxidative stress as important in metabolic and pro-inflammatory signaling. Overall, these data suggested that SDH inhibition with DMM was able to modulate the energetic phenotype and reduce cytokine production in Mos isolated from patients with MASH.

### Energy metabolism pathways in liver MØs

**Figure 6:**
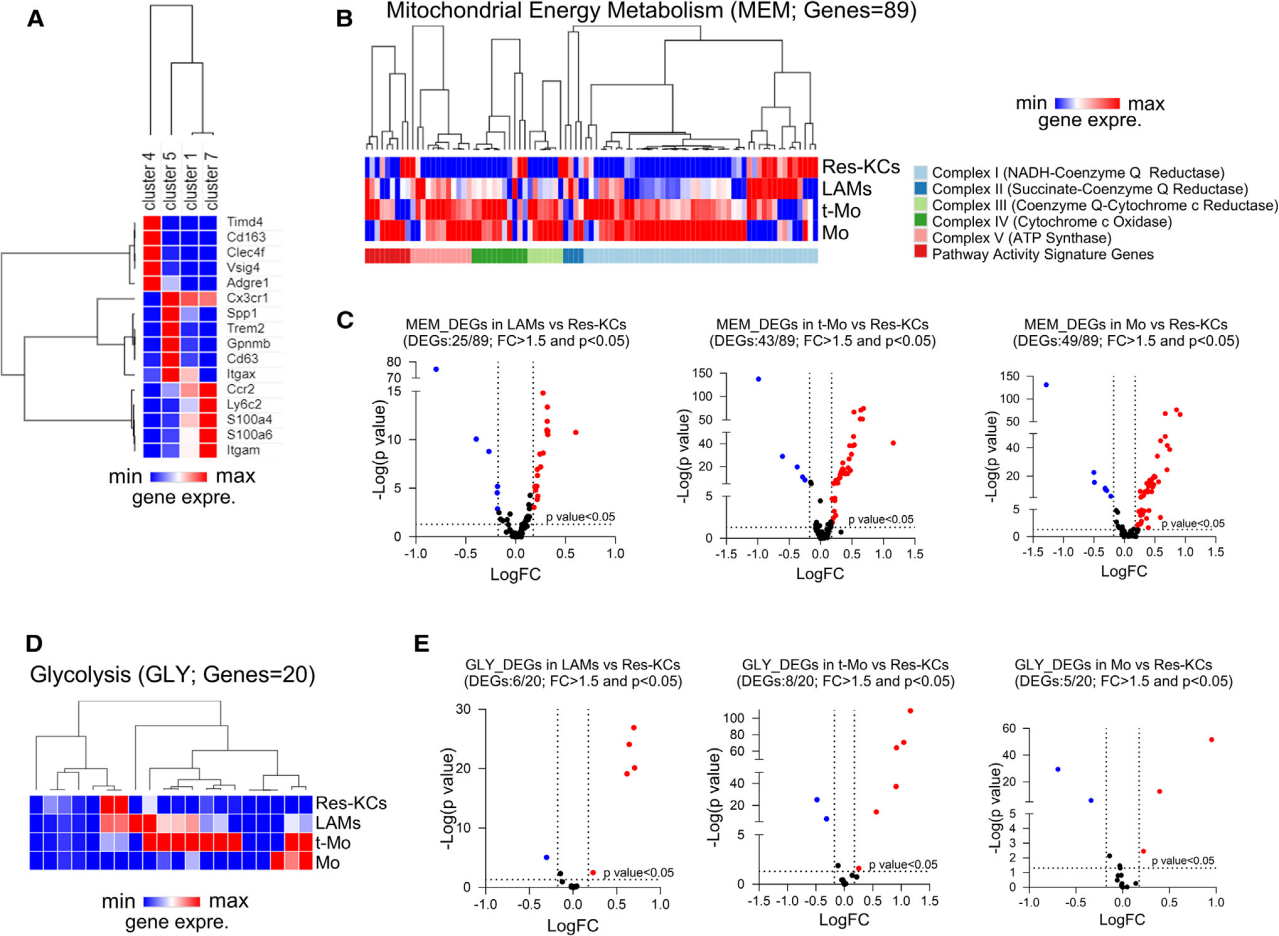
Analysis of scRNA-seq dataset GSE156057 (A) Hierarchical clustering and heatmap of indicated markers in clusters 4, 5, 1, and 7 obtained by clustering analysis of scRNA-seq dataset from mice fed a Western diet for 24 weeks. (B) Hierarchical clustering and heatmap analyses of 89 genes included in MEM pathway. (C) Volcano plot representing MEM DEGs in LAMs vs. Res-KCs, t-Mos vs. Res-KCs, and Mos vs. Res-KCs with a *p* < 0.05 and a fold change (FC) > 1.5. (D) Hierarchical clustering and heatmap analyses of 20 genes included in GLY pathway. (E) Volcano plot representing GLY DEGs in LAMs vs. Res-KCs, t-Mos vs. Res-KCs, and Mos vs. Res-KCs with a *p* < 0.05 and a FC > 1.5. scRNA-seq data for liver CD45^+^ cells in a mouse fed a Western diet for 24 weeks were used from the GEO database (GEO: GSE156057). Differences were detected with Wilcoxon rank-sum test by using ASAPv7 (Swiss Institute of Bioinformatics). MEM, mitochondrial energy metabolism; GLY, glycolysis; DEGs, differentially expressed genes; LAMs, lipid associated macrophages; Mo, monocyte; t-Mo, transitioning-monocyte; Res-KCs, resident Kupffer cells.

Recent studies have demonstrated that during MASH development, KCs are depleted and mostly replaced by Mo-MØs.^26^^,^^27^^,^^28^ Mos infiltrate the liver and differentiate into two major subsets of resident MØs (almost indistinguishable from KCs) and lipid-associated MØs (LAMs).^29^ Here, we analyzed data from the public repository of a single-cell RNA sequencing (scRNA-seq) dataset deposited by Remmerie et al.^27^ in order to assess differences in terms of energy metabolism pathways among Mo-MØ populations. In particular, we dissected data from liver CD45^+^ cells of mice fed a Western diet for 24 weeks, since at this time, the macrophagic scenario is still characterized by the presence of non-inflammatory resident KCs (Res-KCs) and enriches with pro-inflammatory Mos, transitioning populations, and LAMs.^27^ From our analysis, we identified 29 clusters, 9 of which localized in the Mo-MØ portion (based on the expression of *Mafb*, *Ly6c2*, *Fcgr1*, and *Adgre1*) (Figure S4). With the expression of characteristic markers,^27^^,^^29^^,^^30^ we identified cluster 7 as Res-KCs (expressing genes like *Timd4*, *Clec4f*, *Vsig4*, and *Cd163*), cluster 5 as LAMs (expressing genes such as *Spp1*, *Gpnmb*, and *Trem2*), and cluster 7 as Mos (expressing *Ly6c2*), while cluster 1 exhibited an intermediate profile between Mos and LAMs, with high expression of *Ccr2*, and hence is a population of transitioning Mos (t-Mos) likely containing CCR2-dependent LAMs^29^ (Figure 6A). Mo-derived KCs were probably represented in cluster 9, as it included a population of cells expressing *Clec4f* but not *Timd4* (data not shown). Analyzing the mitochondrial energy metabolism (MEM) pathway, we found that Mos, t-Mos, and LAMs presented an upregulated profile compared to Res-KCs (Figure 6B). The volcano plots, showing the MEM differentially expressed genes (DEGs) of the three main infiltrating subsets in comparison with Res-KCs, highlighted that out of 89 genes composing the MEM pathway, LAMs had 25 DEGs (19 upregulated and 6 downregulated), t-Mos had 43 DEGs (38 upregulated and 5 downregulated), and Mos had 49 DEGs (43 upregulated and 6 downregulated) (Figure 6C). In accordance with this, the glycolytic pathway (GLY) was prominently enhanced in Mo-MØs (Figure 6D). As shown by volcano plot, 6 genes out of 20 were significantly upregulated in LAMs, 5 genes in t-Mos, and 3 genes in Mos, against only 1 or 2 downregulated genes (Figure 6E). Collectively, these results underline the enhancement of MEM and GLY pathways in recruited Mos and MØs, along with their already demonstrated pro-inflammatory role compared to normal resident MØs.^27^

These observations were further corroborated by analysis of Seidman et al.’s dataset, RNA-seq performed on sorted MØs of HF-high cholesterol (HC)-fed mice.^28^ Although some contaminations might exist in these sorted cell populations,^27^ we can describe again an upregulation of MEM and GLY in Mo-MØs compared to normal KCs (Figure S5).

### DMM reduces hepatic inflammation in a murine model of MASH

**Figure 7:**
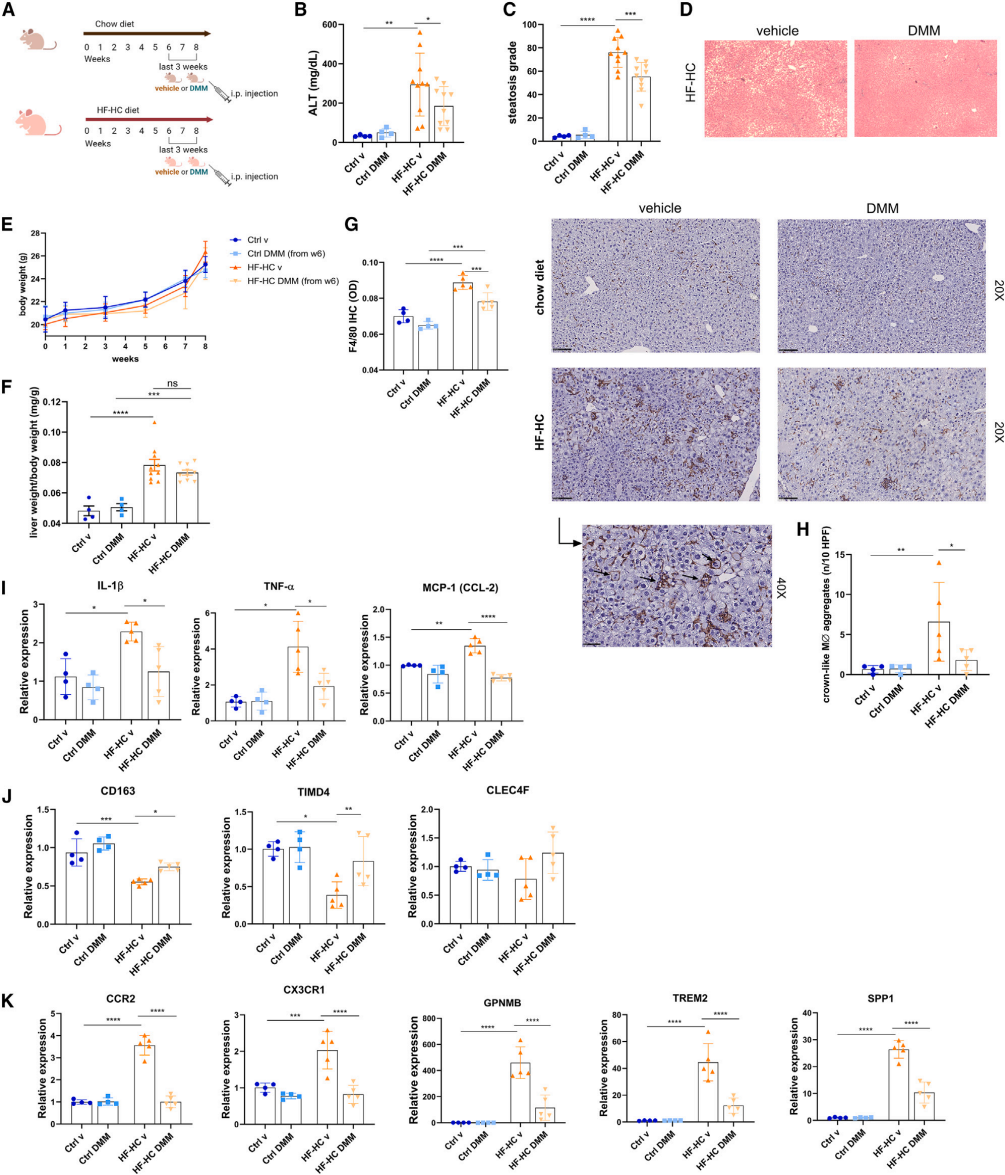
DMM reduces hepatic inflammation in a preclinical model of MASH (A) Experimental design (drawn with BioRender.com). (B) Serum ALT levels (Ctrl groups: *n* = 4; HF-HC groups: *n* = 10). (C and D) Histological determination of hepatic steatosis with representative pictures of H&E staining (Ctrl groups: *n* = 4; HF-HC groups: *n* = 10). (E) Body weight of mice (Ctrl groups: *n* = 4; HF-HC groups: *n* = 10). (F) Liver weight/body weight ratio of mice (Ctrl groups: *n* = 4; HF-HC groups: *n* = 10). (G) Representative pictures and quantification of F4/80 staining in the liver of control and HF-HC mice determined by immunohistochemistry (Ctrl groups: *n* = 4; HF-HC groups: *n* = 5). (H) High-magnification representative picture and quantification of crown-like macrophagic infiltrates determined by F4/80 staining on mice liver (Ctrl groups: *n* = 4; HF-HC groups: *n* = 5). (I) Relative mRNA expression of pro-inflammatory cytokines (*Il-1β* and *Tnf-α*) and MCP-1 (*Ccl-2*) in the liver of control and HF-HC mice determined by qPCR (Ctrl groups: *n* = 4; HF-HC groups: *n* = 5). (J) Relative mRNA expression of KC markers (*Cd163*, *Timd4*, and *Clec4f*) in the liver of control and HF-HC mice determined by qPCR (Ctrl groups: *n* = 4; HF-HC groups: *n* = 5). (K) Relative mRNA expression of Mo-MØ markers (*Ccr2*, *Cx3cr1*, *Gpnmb*, *Trem2*, and *Spp1*) in the liver of control and HF-HC mice determined by qPCR (Ctrl groups: *n* = 4; HF-HC groups: *n* = 5). Data are expressed in mean ± SEM; ∗*p* < 0.05, ∗∗*p* < 0.01, ∗∗∗*p* < 0.001, and ∗∗∗∗*p* < 0.0001 according to one-way ANOVA followed by post hoc analysis (Bonferroni test). IL-1β, interleukin-1β; TNF-α, tumor necrosis factor α; CD163, CD163 antigen; MCP-1 (CCL-2), monocyte chemoattractant protein-1; TIMD4, T cell immunoglobulin and mucin domain containing 4; CLEC4F, C-type lectin domain family 4 member F; CCR2, C-C motif chemokine receptor 2; CX3CR1, C-X3-C motif chemokine receptor 1; GPNMB, glycoprotein Nmb; TREM2, triggering receptor expressed on myeloid cell 2; SPP1, secreted phosphoprotein 1; DMM, dimethyl malonate; HF-HC, high fat and high cholesterol; MØ CLA, macrophagic crown-like aggregate; KCs, Kupffer cells; Mo-MØ, monocyte-derived macrophage.

Considering the hypermetabolic state of circulating Mos in patients with MASH and the possibility of targeting their mitochondrial metabolism and the energy metabolism pathway profiles in Mos and LAMs of murine MASH liver, we investigated the *in vivo* potential effect of DMM in a mouse model of MASH. To do this, wild-type mice were fed an HF-HC diet for 8 weeks to induce steatohepatitis. From week 6, mice were intraperitoneally injected with either DMM at 160 mg/kg or vehicle (PBS) on alternate days. Normal-chow-diet-fed mice served as Ctrl groups (Figure 7A). Interestingly, HF-HC DMM-treated mice presented a significant reduction of cellular damage, as shown by ALT serum levels (Figure 7B) and lower hepatic steatosis (Figures 7C and 7D), compared to vehicle-treated mice, although with no significant change in body weight or liver/body weight ratio (Figures 7E and 7F). As is already known, the addition of cholesterol to the HF diet in mice is efficient to induce a human-like MASH, since both metabolic and inflammatory alterations are well represented.^31^^,^^32^ Moreover, the HF-HC diet induces the recruitment of Mos, enriching the macrophagic population in the liver.^28^^,^^33^ Interestingly, we found an increase of F4/80+ cells in the liver of HF-HC-fed mice, which was significantly counteracted by the administration of DMM (Figure 7G). Moreover, Daemen et al. recently demonstrated that Mo-MØs tend to form crown-like aggregates (CLA),^34^ and here we found that DMM drastically reduced the number of macrophagic CLAs in the liver (Figure 7H). In line with histological features, we found a significant hepatic downregulation of both pro-inflammatory cytokines, *Il-1β* and *Tnf-α*, after DMM treatment, and *Mcp-1* was also reduced by DMM, underlining the importance of this cytokine for Mo recruitment in MASH progression (Figure 7I). In accordance with this, the expression of *Cd163*, *Timd4*, and *Clec4f* demonstrated that KC markers were reduced in HF-HC-fed mice except for the DMM-treated ones (Figure 7J). On the contrary, the expression of Mo-MØ markers, including *Cx3cr1* and *Ccr2*, and LAM markers, including *Gpnmb*, *Trem2*, and *Spp1*, was significantly upregulated in HF-HC vehicle mice and restored to normal levels by DMM administration (Figure 7K). Taken together, our results suggested that the administration of an immunometabolic modulator inhibited the hepatic macrophagic enrichment in a MASH model, reducing inflammation, steatosis, and cellular damage.

## Discussion

The contribution of innate immunity recently emerged as a driving force in the pathogenesis of MASLD and its progression to MASH.^6^^,^^35^ KCs, the resident MØs in the liver and probably the first cells activated by PAMPs and DAMPs, play a key role in the initiation of hepatic inflammation.^36^ However, some studies recently reported the importance of Mos in MASLD progression, as they infiltrate the liver in response to KC depletion and tissue damage, generating a population of Mo-MØs that perpetuate and orchestrate the inflammation. In fact, along the progression of steatosis, the KC population extinguishes, and the liver becomes repopulated by Mos and Mo-MØs.^26^^,^^37^^,^^38^ However, there are still no promising immune interventions, and the global incidence of MASH is growing, constituting a healthcare burden.^2^^,^^39^ Here, we reported the potentialities of a Mo immunometabolism study in MASH. Mos, as well as other immune cells, reprogram their bioenergetic phenotype to support inflammation, as demonstrated in pathological conditions such as autoimmune diseases and critical infections.^40^^,^^41^^,^^42^ On the contrary, very little is known about immunometabolic alterations in chronic and metabolic diseases, such as type 2 diabetes mellitus, cardiovascular diseases, or hepatitis.^43^^,^^44^^,^^45^ Here, we dissected circulating Mo bioenergetics, demonstrating that MASH Mos were characterized by metabolic reprogramming and pro-inflammatory activity. In particular, Mos in patients with MASH presented high levels of glycolysis and elevated mitochondrial respiration associated with dysfunction and oxidative stress. The enzymatic activity of ETC complex I and complex II was indeed significantly enhanced, although it led to high hydrogen peroxide production. In accordance, some authors have previously demonstrated that peripheral blood mononuclear cells (PBMCs) in patients with MASH exhibit oxidative stress.^46^ The production of ROS in leukocytes is an important signaling pathway for the inflammatory activity.^46^ Therefore, it is conceivable to believe that the mitochondrial dysfunction observed in MASH Mos is finalized to the production of pro-inflammatory stimuli, the ROS. Recently, Akhter et al. demonstrated that exposing human PBMCs to hydrogen peroxide promotes the expression of TLR2/4 and the activation of mitogen-activated protein kinase/nuclear factor κB signaling.^47^ Moreover, it has been recently reported that blocking mitochondrial ROS production with MitoTEMPO exerted protective effects in a murine model of MASLD by modulating myeloid-derived suppressor cell recruitment.^43^ Ma et al. suggested instead a role of lipid metabolism and mitochondrial ROS production in CD4^+^ lymphocytes for the progression of MASLD to hepatocellular carcinoma.^48^ Following these observations, we believe that the study of cellular metabolism in leukocytes might be a promising field to explore in MASLD. Here, we decided to focus on Mos because of their central role in inflammatory orchestration, and we tried to target cellular bioenergetics. We have in fact observed that mitochondrial respiration in MASH Mos was sustained by mitochondrial biogenesis as demonstrated by TFAM and PGC-1α induction, and accordingly, several ETC subunits were significantly upregulated. The mechanisms underlying the relationship between inflammatory activation, glycolysis, and mitochondrial activity are very complex and involve biochemical intermediates, HIF proteins, non-coding RNAs, cellular energy sensors, and others.^23^ In particular, we observed a very considerable induction of mTOR in MASH Mos, probably promoted by lower AMPK phosphorylation. The mTOR activity might explain the glycolytic enhancement and, as recently demonstrated, the induction of mitochondrial biogenesis via PGC-1α.^21^^,^^25^ Accordingly, the inhibition of mTOR consistently reduced PGC-1α levels. As already stated, several ETC subunit genes were upregulated, and, in particular, 3 out of 4 *Sdh* subunits (i.e., *Sdha*, *Sdhb*, and *Sdhc*) were more expressed, confirming that the higher activity of complex II was supported at the transcriptional level as well. In line with this, Mills et al. have recently demonstrated that the activity of SDH, also known as complex II, is crucial in promoting metabolic rewiring and inflammatory activity in MØs activated by LPS. The authors showed that the inhibition of SDH restores ETC function, reducing ROS production and HIF-1α activity for the transcription of cytokines and glycolysis genes.^22^ Therefore, we examined the bioenergetic profile of MASH Mos after *in vitro* exposure to DMM, an SDH inhibitor, demonstrating a decrease of both glycolysis and mitochondrial respiration and abrogation of cytokine production. Of note, treatment with DMM restored p-AMPK-mTOR-PGC-1α levels, while the exposure to H_2_O_2_ enhanced the mTOR-PGC-1α activity, confirming a role of this pathway in bioenergetic repurposing.

Overall, we might speculate that circulating PAMPs or DAMPs in MASH activate Mos by inducing a positive feedback mechanism where the increase of mitochondrial activity generates ROSs, which in turn induce mTOR and PGC-1α to transcriptionally sustain glycolysis and mitochondrial respiration, promoting again more oxidative stress. Along these lines, the modulation of mitochondrial metabolism might be the key to control immune activation.

In a second step, we questioned whether metabolic reprogramming could represent a characteristic of liver MØs since, during the development of MASH, the KC population is replaced by Mo-MØs.^26^^,^^28^^,^^34^ Seidman et al. firstly described that myeloid cell diversity in MASH is sustained by Mo recruitment and occupation of different anatomic niches.^28^ More recently, Remmerie et al. and Guilliams et al., through scRNA-seq and spatial proteomics, further characterized a hepatic macrophagic scenario during MASH,^27^^,^^30^ describing several populations and transitional subsets. In MASH murine models, livers enrich with Mos, which differentiate into LAMs and Mo-derived KCs (similar to Res-KCs) passing through transitioning phenotypes.^29^ Analyzing the public scRNA-seq dataset of Remmerie et al.,^27^ we found that Mos, t-Mos, and LAMs exhibited a prominent upregulation of genes included in MEM and glycolysis pathways compared to Res-KCs. This is important, as the inflammatory nature of LAMs compared with KCs is demonstrated,^27^^,^^30^ and the analysis of metabolic pathways suggests that metabolic reprogramming is crucial and probably fuels Mo infiltration and differentiation in the liver.

Therefore, following these results, we finally investigated the potential effect of targeting immunometabolism by using a preclinical model of MASH. We have shown that the injection of DMM exerted immunomodulatory effects *in vivo*, as hepatic inflammation was considerably reduced and the macrophagic enrichment in the liver was inhibited. In particular, the expression of Mo-MØ markers was restored or significantly reduced by DMM, highlighting that targeting cellular bioenergetics contrasted the Mo infiltration and differentiation in MASH livers. Moreover, steatosis and cellular damage were also ameliorated in DMM-treated mice. In conclusion, our findings suggest that targeting Mo immunometabolism in MASH might be considered for a promising therapeutic approach, and research on new modulators of cellular metabolism is needed to open a new field in the treatment of MASH.

### Limitations of the study

Further studies are necessary to find novel molecules able to specifically target Mo or macrophagic subset metabolism. The study highlights the cellular bioenergetic reprogramming in Mos and the possibility of targeting it to improve MASH. However, further studies are needed to highlight specific metabolites involved, and we cannot completely exclude a pleiotropic effect of DMM on other immune cells. In addition to this, although we have excluded a direct effect of DMM on lipid accumulations in hepatocytes (HepG2) *in vitro* (Figure S6), we cannot completely exclude a direct (mild) effect of DMM on hepatic steatosis.

## STAR★Methods

### Key resources table

**Table:** 

REAGENT or RESOURCE	SOURCE	IDENTIFIER
**Antibodies**		

Rabbit polyclonal phospho-mTOR (Ser2448)	Cell signaling Technology	Cat# 2971; RRID: AB_330970
Mouse monoclonal anti-mTOR, Clone L27D4	Cell signaling Technology	Cat# 4517; RRID: AB_1904056
Rabbit monoclonal anti-HIF-1α (D2U3T)	Cell signaling Technology	Cat# 14179; RRID: AB_2622225
Rabbit monoclonal anti-AKT(11E7)	Cell signaling Technology	Cat# 4685; RRID: AB_2225340
Rabbit anti-phospho-Akt (Ser473) (D9E) XP® mAb	Cell signaling Technology	Cat# 4060S; RRID: AB_2315049
Rabbit monoclonal anti-PGC1-α (3G6)	Cell signaling Technology	Cat# 2178; RRID: AB_823600
Rabbit monoclonal anti-AMPK-α, phospho (Thr172), Clone 40H9	Cell signaling Technology	Cat# 2535; RRID: AB_331250
Rabbit polyclonal anti-AMPKα	Cell signaling Technology	Cat# 2532; RRID: AB_330331
Mouse monoclonal anti-β-actin	Sigma-Aldrich	Cat# A1978; RRID: AB_476692
Mouse monoclonal anti-NDUF6B [21C11BC11]	Abcam	Cat# ab110244; RRID: AB_10865349
Mouse monoclonal anti-SDHB [21A11AE7]	Abcam	Cat# ab14714; RRID: AB_301432
Mouse monoclonal anti-ATPB, Clone 3D5	Abcam	Cat# ab14730; RRID: AB_301438
Rabbit monoclonal anti-F4/80 (D2S9R) XP® mAb	Cell signaling Technology	Cat# 70076; RRID: AB_2799771
PE Mouse Anti-Human CD14 Clone MφP9	BD Pharmingen	Cat# 562691; RRID: AB_2737725

**Chemicals, peptides, and recombinant proteins**		

Lipopolysaccaride from *Escherichia coli* O111:B4	Sigma-Aldrich	Cat# LPS25
Dimethyl malonate	Sigma-Aldrich	Cat# 136441; CAS: 108-59-8
Vectastain R.T.U. Elite ABC Reagent	Vector	Cat# PK-7100
Protein Block	Spring Bioscience	Cat# DPB-125
DAKO Liquid DAB+ Substrate Chromogen system	DAKO	Cat# K3468
DRAQ7™ Dye	Thermo Fisher Scientific	Cat# D15106
Oil red O (1-([4-(Xylylazo)xylyl]azo)-2-naphthol, 1-[2,5-Dimethyl-4-(2,5-dimethylphenylazo)phenylazo]-2-naphthol)	Sigma-Aldrich	Cat# O0625; CAS: 1320-06-5
6R-[[(2-chloro-4-fluorophenyl)amino]sulfonyl]-1-cyclohexene-1-carboxylic acid, ethyl ester (TAK-242)	Cayman Chemical	Cat# 13871; CAS: 243984-11-4

**Critical commercial assays**		

Pierce™ Chromogenic Endotoxin Quant Kit	Thermo Fisher Scientific	Cat# A39552
EasySep™ Human CD14 Positive Selection Kit II	Stemcell Techonologies	Cat# 17858
Oxyblot Protein Oxidation Detection Kit	Sigma-Aldrich	Cat# S7150
Human IL-1β ELISA Kit	R&D systems	Cat# DY201
Human TNF-α ELISA Kit	R&D systems	Cat# DY210

**Experimental models: Cell lines**		

HepG2	ATCC	HB-8065

**Experimental models: Organisms/strains**		

Mouse: C57BL6/129SvJ	The Jackson Laboratory	101045

**Oligonucleotides**		

Human Primers	See Table S3	N/A
Mouse Primers	See Table S3	N/A

**Software and algorithms**		

GraphPad Prism 10	GraphPad Software	http://www.graphpad.com.scientific-software/prism/
Agilent Seahorse Analytics	XF Software	https://www.agilent.com/
Fiji software (ImageJ, NIH)	Fiji Software	https://imagej.net/software/fiji/downloads
FlowJo	FlowJo Software	https://www.flowjo.com/
Morpheus	Morpheus Software	https://software.broadinstitute.org/morpheus/
ASAP (Automated Single-cell Analysis Portal)	Swiss Institute of Bioinformatics	https://asap.epfl.ch/
BioRender	BioRender Software	https://www.biorender.com/

Other		

GSE156057	GEO datasets NCBI	https://www.ncbi.nlm.nih.gov/geo/query/acc.cgi?acc=GSE156057
GSE128337	GEO datasets NCBI	https://www.ncbi.nlm.nih.gov/geo/query/acc.cgi?acc=GSE128338

### Resource availability

#### Lead contact

Further information and requests for resources should be requested from the Lead Contact, Dr. Moris Sangineto (email: moris.sangineto@unifg.it).

#### Materials availability

This study did not generate new unique reagents.

#### Data and code availability

• This paper analyzes existing, publicly available data. These accession numbers for the datasets are listed in the key resources table
• This paper does not report original code
• Any additional information required to reanalyze the data reported in this paper is available from the lead contact upon request.

### Experimental model and study participant details

#### CD14^+^ monocytes isolation from PBMCs

26 patients with MASH (determined by liver histology) and 15 age-matched healthy subjects were enrolled for circulating Mo isolation. Baseline characteristics of patients are indicated in Table S2. Exclusion criteria were: oncologic diseases, other concomitant causes of liver diseases (e.g., viral, autoimmune, toxic, alcoholic), autoimmune diseases. Peripheral blood mononuclear cells (PBMCs) were isolated and resuspended in EasySep Buffer (Stemcell Technologies, Grenoble, France). Then, monocytes were purified by using the EasySep Human CD14 Positive Selection Kit II (Stemcell Technologies, Grenoble, France) according to manufacturer’s instructions. Briefly, PBMCs were resuspended in EasySep Buffer, the EasySep EasySep Human CD14 Positive Selection Cocktail II was added to sample for 10 min and then, the EasySep Dextran RapidSpheres 50100, were incubated with the cell suspension for 3 min. After the addition of EasySep Buffer, the suspension was placed into the EasySep Magnet and incubated for 5 min, and to increase purity, this step was repeated twice. CD14^+^ fraction purity was checked by flow cytometry, as shown in Figure S7A. Harvested monocytes were immediately used. All subjects enrolled have signed a letter of informed consent in accordance with the World Medical Association Declaration of Helsinki. Human sample and data collections have been performed in agreement with the guidelines of the Ethics Committee of the “Policlinico Riuniti” of Foggia (permit No. 649/2022) and the European Data Protection Laws.

#### Animal experiments

All the experiments were performed in accordance with the Italian National Laws (DL 116/92) and the European Communities Council Directives (86/609/EEC), after the approvement of the Italian Minister of Health. 6 weeks old male wild type (C57BL/6) mice (from The Jackson Laboratory ©) were maintained at the animal facility of University of Foggia, in individual cages with a 12 h light/12 h dark cycle and fed with chow diet (CD) (Mucedola srl, Milan, Italy) or high fat-high cholesterol diet (HF-HC; 60% cocoa butter +1.25% cholesterol) (Mucedola srl, Milan, Italy) for 8 weeks to induce MASH. From week 6 to the end of experiment (week 8), CD and HF-HC fed mice were divided into two groups and intraperitoneally injected with DMM (160 mg/kg) or vehicle (PBS) on alternate days (Figure 7A), in order to evaluate the effect of DMM *in vivo*. At the end of the study (8 weeks), mice were sacrificed, and blood and liver samples were collected and stored at −80°C or fixed in 10%-buffered formalin for histology.

#### Cell lines

Human hepatocellular carcinoma cells (HepG2), obtained from American Type Culture Collection (ATCC), were cultured in Dulbecco’s modified Eagle’s medium (DMEM) (Gibco) supplemented with 10% heat-inactivated (FBS) (Gibco) and 1% of P/S (Sigma Aldrich) at 37°C and 5% CO_2_. All cells were grown under 5% CO_2_ at 37°C. Cell cultures were checked for mycoplasma contamination using the MycoStrip Mycoplasma detection kit (Invivogen).

### Method details

#### Isolation of peripheral blood mononuclear cells

Peripheral blood mononuclear cells (PBMCs) were immediately isolated from peripheral venous EDTA-blood of MASH patients (*n* = 26) and healthy controls (*n* = 15) using Lymphoprep (Stemcell Technologies, Grenoble, France) with density gradient centrifugation according to the manufacturer’s procedure. Briefly, 20 mL of blood were diluted to a 1:1 volume ratio with the EasySep Buffer (Stemcell Technologies, Grenoble, France), layered on top of 15 mL of Lymphoprep and centrifuged at 1200xg for 10 min at 25°C. The harvested mononuclear cells were collected, resuspended in EasySep Buffer and washed twice in the same buffer, with sequential centrifugation, at 250xg for 10min and 200xg for 10min, respectively. Finally, the pellet was freshly used for isolation of CD14^+^ monocytes (Mo).

#### CD14^+^ Mo stimulation

CD14^+^ Mo were plated in 6 well plates and in Seahorse XF HS Miniplates (Agilent Technologies) precoated with poly-L-lysine, at density of 2x10^5^ and 1x10^5^ cells per each well, respectively, and exposed to DMM (10 mM) or PBS for 4 h, prior to perform bioenergetics analysis and RNA isolation for expression studies. The concentration of DMM was based on a previous study conducted on monocytes,^49^ after having excluded negative effects on cell vitality with trypan blue staining and living cell count with TC10 Automated Cell Counter (Bio Rad Laboratories Inc, Segrate (MI), Italy) (Figure S7B). To evaluate the contribution of circulating factors to Mo activation, CD14^+^ Mo of healthy controls were plated in RPMI medium (Gibco) supplemented with 10% heat-inactivated fetal bovine serum (FBS; Gibco), and 1% penicillin and streptomycin (P/S; Sigma Aldrich) for 1h to allow the attachment; after 1 h medium was replaced with fresh RPMI supplemented with 10% MASH or healthy controls serum (as a replacement of FBS) for 4h. Moreover, serum-treated cells were pre-exposed to TAK-242 (Cayman chemical), a specific inhibitor of TLR4 signaling, at 1 μM for 1 h to study the involvement of TLR4. In another experimental setting, CD14^+^ Mo of healthy controls were stimulated with LPS (*Escherichia coli* O111:B4, Sigma-Aldrich) (50 ng/mL).

To study the role of mTOR to induce PGC-1α, MASH Mo were treated with everolimus (Stem Cell Techonolgies), a selective mTOR inhibitor, at the concentration of 10nM for 4 h.

Concentrations of LPS, TAK-242 and everolimus were based on previous authoritative studies.^50^^,^^51^^,^^52^

#### Bioenergetic studies

To measure glycolysis and mitochondrial respiration, proton efflux rate (PER) and oxygen consumption rate (OCR) were respectively quantified by using the Seahorse XF HS Mini Analyzer (Agilent Technologies) according to the manufacturer’s instructions. The cartridge plate was hydrated with sterile water and incubated overnight (37°C, CO_2_-free); the assay medium (Seahorse XF RPMI assay medium, pH 7.4 containing 1 mM pyruvate, 2 mM glutamine and 10 mM glucose) was prepared immediately before assay. CD14^+^ Mo were freshly isolated from healthy subjects and MASH patients and analyzed the same day on different runs, loading 1–2 Ctrls with 1–2 MASH samples simultaneously on each plate. 1 x 10^5^ Mo/well were plated in duplicate on the XF HS Mini cell culture microplate precoated with poly-L-lysine. Glycolytic activity was monitored by measuring PER after sequential injection rotenone/antimycin A and 2-deoxy-glucose (2-DG) by using the “Seahorse XF Glycolytic Rate Assay Kit” (Agilent Technologies). OCR was measured under basal conditions as well as after three serial injections of oligomycin, FCCP and rotenone/antimycin A by using the “Seahorse XF Cell Mito Stress Test kit” (Agilent Technologies). Analysis was performed with Agilent Seahorse Analytics software and data were normalized to total cellular protein using BCA protein assay (Thermo Fisher Scientific), reporting protein concentration directly to Agilent Seahorse Analytics software for automatic normalization.

#### Mitochondrial respiratory chain complexes activity

The mitochondria-enriched fractions from cell suspension were prepared as previously reported^23^^,^^31^ with minor adjustments. Briefly, cells were washed with ice-cold phosphate-buffered saline medium and centrifuged at 3500 rpm for 6 min at 4°C. Supernatant was removed and the cell pellet was resuspended in 10 mM/L Tris, pH 7.6, with protease and phosphatase inhibitors (Sigma-Aldrich), freeze thawed thrice in liquid nitrogen. Cells were also mechanically disrupted with a 2-mL glass/Teflon potter on ice. The concentration of mitochondria enriched cell suspension was estimated by Bradford assay. The enzymatic activities of mitochondrial complexes were performed spectrophotometrically, by following variations in the UV-VIS absorbance of colorimetric substrates or reaction products by using NanoDrop One (Thermo Fisher Scientific).

#### Complex I assay

Complex I was measured spectrophotometrically at 600 nm as previously reported.^53^ 35 μg of each enzyme solution was mixed with complex I buffer (25 mM/L potassium phosphate, 3.5 g/L fatty acid free BSA), 60 μM/L 2,6-Dichlorophenolindophenol (DCIP), 70 μM/L decylubiquinone (DBH), 1.0 μM/L antimycin-A) and incubated for 3 min at 37°C. Then, we added 10 mM/L NADH and measured the absorbance for 4 min at 37°C. Later the reference was measured in the presence of 2.5 μM rotenone. Enzyme activity was calculated with molar extinction coefficient (ε) for the DCPIP (19.1 mM^−1^ cm^−1^).

#### Complex II assay

Complex II was measured spectrophotometrically at 600 nm as previously reported.^54^ 40 μg of each cell suspension was incubated with complex II buffer (25 mM KH2PO4 (pH 7.8), 2 mM EDTA, 1 mg/mL fatty acid free BSA), 10 mM succinate, 50uM DBH, 1 mM KCN, 4 μM rotenone, and 10 μM antimycin A, 0.2 mM ATP) for 10 min. After the addition of 80 μM DCIPIP, the change in absorbance at 600 nm was recorded for 2 min for reference. The addition of 10 mM malonate inhibits the oxidation of succinate. Enzyme activity was calculated with ε for the DBH (16 mM^−1^ cm^−1^).

#### Measurement of mitochondrial H_2_O_2_ production

The rate of peroxide production was determined in human Mo. H_2_O_2_ production was measured at 37°C following the oxidation of Amplex Red by horseradish peroxidase using 5 mM pyruvate plus 1 mM malate or 5 mM succinate as respiratory substrates. The fluorescence of supernatants was measured using 530 nm as excitation wavelength and 590 nm as emission wavelength in filter max F5 multimode microplate reader (Beckman Coulter; DTX 880 Multimode Detector). The rate of peroxide production was calculated using a standard curve of H_2_O_2_.

#### Circulating LPS measurement

Circulating LPS in serum of MASH patients (*n* = 10) and healthy controls (*n* = 7) was determined using Pierce Chromogenic Endotoxin Quant Kit (Thermo Fisher scientific), an endpoint amebocyte lysate assay that quantifies endotoxins (LPS). According to the manufacturer’s protocol, serum was collected from blood, centrifuged at 1300g for 15 min and then stored at −20°C. Serum samples were 1:50 diluted with endotoxin-free water and subsequently heated to 70°C for 15 min to inactivate inhibitory proteins. The amount of LPS was quantified by the addition of a chromogenic substrate; the activated protease, catalyzed the release of *p*-nitroaniline (pNA), spectrophotometrically detected at 405 nm in filter max F5 multimode microplate reader (Beckman Coulter; DTX 880 Multimode Detector). LPS levels (EU/mL) were calculated based on the standard curve.

#### Expression studies

Total RNA was isolated from human monocytes and mice livers using QIAzol Lysis Reagent (Qiagen). Equal amounts of RNA were reverse transcribed to cDNA using a high-capacity cDNA reverse transcription kit (Applied Biosystems) according to the manufacturer’s instructions. Real-time PCR was performed, using Sso Advanced universal SYBR green supermix on a Bio-Rad CFX96 Real-Time system as previously reported,^23^ using primers listed in Table S3 and PrimePCR array “Mitochondria Energy Metabolism Plus” (Bio-Rad Laboratories Inc, Segrate (MI), Italy). The cycle threshold (Ct) was determined, and the relative gene expression was calculated with the ΔΔCT method. The gene expression was normalized to human or mouse β-actin.

#### External datasets analysis

ScRNA-seq dataset for liver CD45^+^ cells from a mouse fed a western-diet for 24 weeks was downloaded from GEO dataset (GSE156057).^27^ Processing of data was done by using the web-based platform ASAP (Swiss Institute of Bioinformatics).^55^ Following the Seurat pipeline of ASAP, outlier cells were discarded based on 3 parameters (UMI/reads and number of expressed genes). Then counts were normalized with the LogNormalize Seurat function. High variable genes (HVG) were found with the Dispersion Seurat function selecting 2000 top variable features. The Scaling Seurat function was used to scale and center features. The Principal Component Analysis, clustering and UMAP were performed using the Seurat pipeline. For the clustering, 50 principal components were used from PCA and 0.8 was used as the resolution parameter. Differential gene expression was assessed using the Wilcoxon Rank-Sum Test. Differentially expressed genes (DEGs) were determined by *p* value < 0.05 and fold change > 1.5.

We selected a set of genes representing key metabolic proteins in Glycolysis and Mitochondrial energy metabolism (identified by searching the SBI databases; see Table S4). Hierarchical clustering and heatmap analyses were performed using a Versatile matrix visualization and analysis software Morpheus (https://software.broadinstitute.org/morpheus/).

Another analysis was conducted on normalized RNA-seq data for different Myeloid Cell populations in HF-HC fed mice, downloaded from GEO database (GSE128337).^28^

Differentially expressed genes (DEGs) in Glycolysis and Mitochondrial energy metabolism were determined by *p* value < 0.05 (with two-tailed Student’s t test) and fold change >1.5 (Table S5). Hierarchical clustering and heatmap analyses were performed as described above.

#### Flow cytometry

A PE mouse anti-human CD14 antibody (BD Pharmigen) was used to detect the purity of cells isolated by CD14 positive selection (Figure S5). We performed FACS analysis on Canto2 cytometers (Becton Dickinson). We analyzed flow cytometry data using FlowJo software (Becton Dickinson). Cell viability was measured by DRAQ7 dye (Thermo Fisher Scientific) staining exclusion.

#### Western blot analysis

Lysates from monocytes with equal amounts of protein (30 μg) were loaded in 8%, 10% or 12% SDS-PAGE, transferred to a nitrocellulose membrane (Bio-Rad Laboratories Inc, Segrate (MI), Italy) and blocked with 5% bovine serum albumin (BSA) in Tris-buffered saline containing 0.1% Tween 20 (TBST) for 1 h at room temperature. Then, membranes were incubated overnight at 4°C with these following primary antibodies: Complex I subunit NDUFB6 monoclonal antibody (Abcam, ab110244), Complex II subunit SDHB monoclonal antibody (Abcam, ab14714), Anti-ATPB antibody (3D5) - Mitochondrial Marker (Complex V beta subunit) (Abcam, ab14730), mTOR (1:1000, Cell Signaling Technology), *p*-mTOR (1:1000, Cell Signaling Technology), PGC-1α (1:1000, Cell Signaling Technology), AMPK (1:1000, Cell Signaling Technology), *p*-AMPK (1:1000, Cell Signaling Technology), AKT (1:1000, Cell Signaling Technology), *p*-AKT (Cell Signaling Technology, 1:1000), HIF-1α (1:1000, Cell Signaling Technology) and β-actin (1:1000, Thermo Fisher Scientific) as loading control. Therefore, membranes were incubated with the appropriate HRP-conjugated secondary antibody (1:2000, Cell Signaling Technology) for 1 h at room temperature and bands were detected by the Clarity Western ECL Blotting Substrate using a ChemiDoc MP system (Bio-Rad Laboratories Inc, Segrate (MI), Italy) and quantified by the Image Lab Software.

#### Histology

Sections of formalin-fixed, paraffin-embedded mice liver samples were stained with haematoxylin/eosin and blind-analysed by a pathologist in order to quantify hepatic steatosis by calculating the percentage of cells with macrovesicular and microvesicular steatosis, as described in a previous work.^56^^,^^57^

#### F4/80 immunohistochemistry

Mice liver sections were stained as previously described.^56^ Briefly, after deparaffinization of slides in xylene and dehydration in an ethanol gradient, the antigen unmasking with sodium-citrate buffer (pH = 6) was performed. To quench endogenous peroxidase activity, sections were incubated with peroxidase blocking reagent (3% H_2_O_2_ in methanol) for 10 min and rinsed with H_2_O for 5 min. Then a protein blocking was executed with Protein Block ready to use (Spring Bioscience). Primary antibody was rabbit anti-F4/80 (Cell Signaling Technology), while the secondary antibody was a Biotinylated anti-rabbit antibody (Vector Laboratories, Inc.). Finally, the sections were incubated for 30 min with R.T.U. Vectastain, stained with DAB (Dako, Santa Clara, CA) and counterstained with hematoxylin (Leica Biosystem). The F4/80 positivity was quantified with Fiji software (ImageJ, NIH) and subsequent conversion in optical density (OD). A pathologist counted the number of F4/80+ cell aggregates in at least 10 high power field.

#### Western blot analysis of oxidized proteins

The analysis of oxidized proteins was performed by western blot using an Oxyblot Protein Oxidation Detection kit (Sigma-Aldrich), as previously described.^32^ The same amounts of proteins (25 μg) were reacted with dinitrophenylhydrazine (DNPH) for 20 min, followed by neutralization with a solution containing glycerol and 2-mercaptoethanol, resolved in 12% SDS-polyacrylamide gel electrophoresis. After the transfer to a nitrocellulose membrane, a blocking step with 1% BSA and incubation with a rabbit anti-DNPH antibody (1: 150) at 4°C overnight followed. After washing, the membrane was incubated with the secondary antibody (1:2000, Cell Signaling Technology) conjugated to horseradish peroxidase and detected by Clarity Western ECL Blotting Substrate using a ChemiDoc MP system (Bio-Rad Laboratories Inc, Segrate (MI), Italy). The test provides a qualitative analysis of the total protein oxidation state change.

#### Enzyme-linked immunosorbent assay (ELISA)

The concentrations of IL-1β and TNF-α were measured in Mo supernatants and serum by an ELISA kit, (R&D Systems, Minneapolis, MN, USA). Briefly, according to the assay protocol, 96-well microplates were coated with 100 μL of specific capture antibody and incubated overnight at room temperature. The microplates were washed three times with a wash buffer (0.05% Tween 20 in PBS) and blocked with 300 μL per well of blocking reagent (1% BSA in PBS, pH 7.0–7.2) 1 h at room temperature; after three washes, the samples and standards were added to each well and incubated for 2 h at room temperature. A biotin-conjugated detection antibody was added to each well, incubated for 2 h; after three washes, 100 μL of Streptavidin-HRP were added and incubated in the dark for 20 min and then, 100 μL of a Substrate Solution (1:1 mixture of H_2_O_2_ and tetramethylbenzidine), incubated in the dark for 20 min. The reaction was stopped by the addition of 50 μL of a Stop Solution (2N H_2_SO_4_) to each well and the absorbance was measured at 450 nm using a filter max F5 multimode microplate reader (Beckman Coulter; DTX 880 Multimode Detector).

#### HepG2 stimulation and Oil Red O staining

HepG2 (4x10^5^ cells/well) were plated in 6 well-plates in DMEM complete medium for 24 h to allow attachment. After 24h medium and unattached cells were removed and replaced with fresh DMEM and supplemented with 8% intralipid (Baxter) to induce the steatotic condition and simultaneously treated with DMM (10 mM and 20 mM) for 48 h. To measure lipid droplets accumulation, cells were stained with Oil Red O (ORO). Briefly, cells were rinsed in PBS, fixed in 4% paraformaldehyde for 1 h, washed twice with ddH_2_O and permeabilized with 60% isopropyl alcohol for 5 min. Then, cells were dried and stained with Oil Red O for 30 min at room temperature, rinsed with ddH_2_O and photographed. Staining intensity was measured by Fiji software (ImageJ) and converted to optical density (OD). To quantify lipid accumulation, ddH_2_O was removed, cells were completely dried and Oil Red O dye was eluted by adding 100% isopropanol, incubated for 10 min with gently shaking. The isopropanol with Oil Red O was pipetted up and down several times and absorption at 500 nm was measured using 100% isopropanol as blank, with NanoDrop One (Thermo Fisher Scientific).

### Quantification and statistical analysis

GraphPad PRISM 10 was used for statistical analysis. Unpaired two-tailed Student’s t test and one-way analysis of variance followed by post hoc Bonferroni test were used when appropriate. Results are shown as mean ± standard error of mean (SEM). Details of the statistical analyses (*n* of samples, *n* of experiments, and test used) can be found in the main text and figure captions. Statistical significance thresholds were set at ∗*p* < 0.05, ∗∗*p* < 0.01, ∗∗∗*p* < 0.001, ∗∗∗∗*p* < 0.0001.

## Supplemental information

Document S1. Figures S1–S7 and Tables S1–S3

Table S4. DEGS from GSE156057, related to Figure 6

Table S5. DEGs from GSE128337, related to external datasets analysis

Document S2. Article plus supplemental information

## References

[1] Anstee Q.M., Targher G., Day C.P. (2013). Progression of NAFLD to diabetes mellitus, cardiovascular disease or cirrhosis. Nat. Rev. Gastroenterol. Hepatol..

[2] Witkowski M., Moreno S.I., Fernandes J., Johansen P., Augusto M., Nair S. (2022). The Economic Burden of Non-Alcoholic Steatohepatitis: A Systematic Review. Pharmacoeconomics.

[3] Hardy T., Oakley F., Anstee Q.M., Day C.P. (2016). Nonalcoholic Fatty Liver Disease: Pathogenesis and Disease Spectrum. Annu. Rev. Pathol..

[4] Noureddin M., Mato J.M., Lu S.C. (2015). Nonalcoholic fatty liver disease: update on pathogenesis, diagnosis, treatment and the role of S-adenosylmethionine. Exp. Biol. Med..

[5] Buzzetti E., Pinzani M., Tsochatzis E.A. (2016). The multiple-hit pathogenesis of non-alcoholic fatty liver disease (NAFLD). Metabolism..

[6] Sutti S., Albano E. (2020). Adaptive immunity: an emerging player in the progression of NAFLD. Nat. Rev. Gastroenterol. Hepatol..

[7] Dallio M., Sangineto M., Romeo M., Villani R., Romano A.D., Loguercio C., Serviddio G., Federico A. (2021). Immunity as Cornerstone of Non-Alcoholic Fatty Liver Disease: The Contribution of Oxidative Stress in the Disease Progression. Int. J. Mol. Sci..

[8] Weismann D., Binder C.J. (2012). The innate immune response to products of phospholipid peroxidation. Biochim. Biophys. Acta.

[9] Krenkel O., Tacke F. (2017). Macrophages in Nonalcoholic Fatty Liver Disease: A Role Model of Pathogenic Immunometabolism. Semin. Liver Dis..

[10] Parthasarathy G., Malhi H. (2021). Macrophage Heterogeneity in NASH: More Than Just Nomenclature. Hepatology.

[11] Lachmandas E., Boutens L., Ratter J.M., Hijmans A., Hooiveld G.J., Joosten L.A., Rodenburg R.J., Fransen J.A., Houtkooper R.H., van Crevel R. (2016). Microbial stimulation of different Toll-like receptor signalling pathways induces diverse metabolic programmes in human monocytes. Nat Microbiol.

[12] Van den Bossche J., O'Neill L.A., Menon D. (2017). Macrophage Immunometabolism: Where Are We (Going)?. Trends Immunol..

[13] Marrocco A., Ortiz L.A. (2022). Role of metabolic reprogramming in pro-inflammatory cytokine secretion from LPS or silica-activated macrophages. Front. Immunol..

[14] Martínez-Reyes I., Chandel N.S. (2020). Mitochondrial TCA cycle metabolites control physiology and disease. Nat. Commun..

[15] Hotamisligil G.S. (2017). Foundations of Immunometabolism and Implications for Metabolic Health and Disease. Immunity.

[16] Gilgenkrantz H., Mallat A., Moreau R., Lotersztajn S. (2021). Targeting cell-intrinsic metabolism for antifibrotic therapy. J. Hepatol..

[17] Shadel G.S., Horvath T.L. (2015). Mitochondrial ROS signaling in organismal homeostasis. Cell.

[18] Ekstrand M.I., Falkenberg M., Rantanen A., Park C.B., Gaspari M., Hultenby K., Rustin P., Gustafsson C.M., Larsson N.G. (2004). Mitochondrial transcription factor A regulates mtDNA copy number in mammals. Hum. Mol. Genet..

[19] Scarpulla R.C. (2011). Metabolic control of mitochondrial biogenesis through the PGC-1 family regulatory network. Biochim. Biophys. Acta.

[20] Wang S., Liu R., Yu Q., Dong L., Bi Y., Liu G. (2019). Metabolic reprogramming of macrophages during infections and cancer. Cancer Lett..

[21] Cunningham J.T., Rodgers J.T., Arlow D.H., Vazquez F., Mootha V.K., Puigserver P. (2007). mTOR controls mitochondrial oxidative function through a YY1-PGC-1alpha transcriptional complex. Nature.

[22] Mills E.L., Kelly B., Logan A., Costa A.S.H., Varma M., Bryant C.E., Tourlomousis P., Däbritz J.H.M., Gottlieb E., Latorre I. (2016). Succinate Dehydrogenase Supports Metabolic Repurposing of Mitochondria to Drive Inflammatory Macrophages. Cell.

[23] Sangineto M., Ciarnelli M., Cassano T., Radesco A., Moola A., Bukke V.N., Romano A., Villani R., Kanwal H., Capitanio N. (2023). Metabolic reprogramming in inflammatory microglia indicates a potential way of targeting inflammation in Alzheimer's disease. Redox Biol..

[24] Yang Y., Shao R., Tang L., Li L., Zhu M., Huang J., Shen Y., Zhang L. (2019). Succinate dehydrogenase inhibitor dimethyl malonate alleviates LPS/d-galactosamine-induced acute hepatic damage in mice. Innate Immun..

[25] Yu Q., Wang Y., Dong L., He Y., Liu R., Yang Q., Cao Y., Wang Y., Jia A., Bi Y., Liu G. (2020). Regulations of Glycolytic Activities on Macrophages Functions in Tumor and Infectious Inflammation. Front. Cell. Infect. Microbiol..

[26] Devisscher L., Scott C.L., Lefere S., Raevens S., Bogaerts E., Paridaens A., Verhelst X., Geerts A., Guilliams M., Van Vlierberghe H. (2017). Non-alcoholic steatohepatitis induces transient changes within the liver macrophage pool. Cell. Immunol..

[27] Remmerie A., Martens L., Thoné T., Castoldi A., Seurinck R., Pavie B., Roels J., Vanneste B., De Prijck S., Vanhockerhout M. (2020). Osteopontin Expression Identifies a Subset of Recruited Macrophages Distinct from Kupffer Cells in the Fatty Liver. Immunity.

[28] Seidman J.S., Troutman T.D., Sakai M., Gola A., Spann N.J., Bennett H., Bruni C.M., Ouyang Z., Li R.Z., Sun X. (2020). Niche-Specific Reprogramming of Epigenetic Landscapes Drives Myeloid Cell Diversity in Nonalcoholic Steatohepatitis. Immunity.

[29] Barreby E., Chen P., Aouadi M. (2022). Macrophage functional diversity in NAFLD - more than inflammation. Nat. Rev. Endocrinol..

[30] Guilliams M., Bonnardel J., Haest B., Vanderborght B., Wagner C., Remmerie A., Bujko A., Martens L., Thoné T., Browaeys R. (2022). Spatial proteogenomics reveals distinct and evolutionarily conserved hepatic macrophage niches. Cell.

[31] Bellanti F., Villani R., Tamborra R., Blonda M., Iannelli G., di Bello G., Facciorusso A., Poli G., Iuliano L., Avolio C. (2018). Synergistic interaction of fatty acids and oxysterols impairs mitochondrial function and limits liver adaptation during nafld progression. Redox Biol..

[32] Sangineto M., Bukke V.N., Bellanti F., Tamborra R., Moola A., Duda L., Villani R., Romano A.D., Serviddio G. (2021). A Novel Nutraceuticals Mixture Improves Liver Steatosis by Preventing Oxidative Stress and Mitochondrial Dysfunction in a NAFLD Model. Nutrients.

[33] McGettigan B., McMahan R., Orlicky D., Burchill M., Danhorn T., Francis P., Cheng L.L., Golden-Mason L., Jakubzick C.V., Rosen H.R. (2019). Dietary Lipids Differentially Shape Nonalcoholic Steatohepatitis Progression and the Transcriptome of Kupffer Cells and Infiltrating Macrophages. Hepatology.

[34] Daemen S., Gainullina A., Kalugotla G., He L., Chan M.M., Beals J.W., Liss K.H., Klein S., Feldstein A.E., Finck B.N. (2021). Dynamic Shifts in the Composition of Resident and Recruited Macrophages Influence Tissue Remodeling in NASH. Cell Rep..

[35] Huby T., Gautier E.L. (2022). Immune cell-mediated features of non-alcoholic steatohepatitis. Nat. Rev. Immunol..

[36] Kazankov K., Jørgensen S.M.D., Thomsen K.L., Møller H.J., Vilstrup H., George J., Schuppan D., Grønbæk H. (2019). The role of macrophages in nonalcoholic fatty liver disease and nonalcoholic steatohepatitis. Nat. Rev. Gastroenterol. Hepatol..

[37] Bonnardel J., T'Jonck W., Gaublomme D., Browaeys R., Scott C.L., Martens L., Vanneste B., De Prijck S., Nedospasov S.A., Kremer A. (2019). Stellate Cells, Hepatocytes, and Endothelial Cells Imprint the Kupffer Cell Identity on Monocytes Colonizing the Liver Macrophage Niche. Immunity.

[38] Neuschwander-Tetri B.A. (2018). Pharmacologic Management of Nonalcoholic Steatohepatitis. Gastroenterol. Hepatol..

[39] Fukui S., Iwamoto N., Takatani A., Igawa T., Shimizu T., Umeda M., Nishino A., Horai Y., Hirai Y., Koga T. (2017). M1 and M2 Monocytes in Rheumatoid Arthritis: A Contribution of Imbalance of M1/M2 Monocytes to Osteoclastogenesis. Front. Immunol..

[40] Gibellini L., De Biasi S., Paolini A., Borella R., Boraldi F., Mattioli M., Lo Tartaro D., Fidanza L., Caro-Maldonado A., Meschiari M. (2020). Altered bioenergetics and mitochondrial dysfunction of monocytes in patients with COVID-19 pneumonia. EMBO Mol. Med..

[41] Maher A.K., Burnham K.L., Jones E.M., Tan M.M.H., Saputil R.C., Baillon L., Selck C., Giang N., Argüello R., Pillay C. (2022). Transcriptional reprogramming from innate immune functions to a pro-thrombotic signature by monocytes in COVID-19. Nat. Commun..

[42] Zhang I.W., Curto A., López-Vicario C., Casulleras M., Duran-Güell M., Flores-Costa R., Colsch B., Aguilar F., Aransay A.M., Lozano J.J. (2022). Mitochondrial dysfunction governs immunometabolism in leukocytes of patients with acute-on-chronic liver failure. J. Hepatol..

[43] Garcia C.C., Piotrkowski B., Baz P., Poncino D., Benavides J., Colombato L., Toso M.L.R., Yantorno S., Descalzi V., Gondolesi G.E. (2022). A Decreased Response to Resistin in Mononuclear Leukocytes Contributes to Oxidative Stress in Nonalcoholic Fatty Liver Disease. Dig. Dis. Sci..

[44] Villani R., Sangineto M., Pontrelli P., Bellanti F., Bukke V.N., Moola A., Gesualdo L., Vendemiale G., Grandaliano G., Stallone G. (2022). Eradication of HCV by direct antiviral agents restores mitochondrial function and energy homeostasis in peripheral blood mononuclear cells. Faseb. J..

[45] Yvan-Charvet L., Bonacina F., Guinamard R.R., Norata G.D. (2019). Immunometabolic function of cholesterol in cardiovascular disease and beyond. Cardiovasc. Res..

[46] Mittal M., Siddiqui M.R., Tran K., Reddy S.P., Malik A.B. (2014). Reactive oxygen species in inflammation and tissue injury. Antioxidants Redox Signal..

[47] Akhter N., Madhoun A., Arefanian H., Wilson A., Kochumon S., Thomas R., Shenouda S., Al-Mulla F., Ahmad R., Sindhu S. (2019). Oxidative Stress Induces Expression of the Toll-Like Receptors (TLRs) 2 and 4 in the Human Peripheral Blood Mononuclear Cells: Implications for Metabolic Inflammation. Cell. Physiol. Biochem..

[48] Ma C., Zhang Q., Greten T.F. (2018). Nonalcoholic fatty liver disease promotes hepatocellular carcinoma through direct and indirect effects on hepatocytes. FEBS J..

[49] Codo A.C., Davanzo G.G., Monteiro L.B., de Souza G.F., Muraro S.P., Virgilio-da-Silva J.V., Prodonoff J.S., Carregari V.C., de Biagi Junior C.A.O., Crunfli F. (2020). Elevated Glucose Levels Favor SARS-CoV-2 Infection and Monocyte Response through a HIF-1alpha/Glycolysis-Dependent Axis. Cell Metabol..

[50] Pang T., Wang J., Benicky J., Saavedra J.M. (2012). Minocycline ameliorates LPS-induced inflammation in human monocytes by novel mechanisms including LOX-1, Nur77 and LITAF inhibition. Biochim. Biophys. Acta.

[51] Owens A.P., Passam F.H., Antoniak S., Marshall S.M., McDaniel A.L., Rudel L., Williams J.C., Hubbard B.K., Dutton J.A., Wang J. (2012). Monocyte tissue factor-dependent activation of coagulation in hypercholesterolemic mice and monkeys is inhibited by simvastatin. J. Clin. Invest..

[52] Baetta R., Granata A., Canavesi M., Ferri N., Arnaboldi L., Bellosta S., Pfister P., Corsini A. (2009). Everolimus inhibits monocyte/macrophage migration in vitro and their accumulation in carotid lesions of cholesterol-fed rabbits. J. Pharmacol. Exp. Therapeut..

[53] Pollard A.K., Craig E.L., Chakrabarti L. (2016). Mitochondrial Complex 1 Activity Measured by Spectrophotometry Is Reduced across All Brain Regions in Ageing and More Specifically in Neurodegeneration. PLoS One.

[54] Barrientos A. (2002). In vivo and in organello assessment of OXPHOS activities. Methods.

[55] Gardeux V., David F.P.A., Shajkofci A., Schwalie P.C., Deplancke B. (2017). ASAP: a web-based platform for the analysis and interactive visualization of single-cell RNA-seq data. Bioinformatics.

[56] Sangineto M., Grabherr F., Adolph T.E., Grander C., Reider S., Jaschke N., Mayr L., Schwärzler J., Dallio M., Moschen A.R. (2020). Dimethyl fumarate ameliorates hepatic inflammation in alcohol related liver disease. Liver Int..

[57] Sangineto M., Grander C., Grabherr F., Mayr L., Enrich B., Schwärzler J., Dallio M., Bukke V.N., Moola A., Moschetta A. (2022). Recovery of Bacteroides thetaiotaomicron ameliorates hepatic steatosis in experimental alcohol-related liver disease. Gut Microb..

