# Supplementary material for: Monocyte bioenergetics: An immunometabolic perspective in metabolic dysfunction-associated steatohepatitis
Source: Cell Rep Med. 2024 May 10;5(5):101564. doi: 10.1016/j.xcrm.2024.101564 (PMC11148801; doi:10.1016/j.xcrm.2024.101564)
Supplement: Document S1. Figures S1–S7 and Tables S1–S3 [file mmc1.pdf]

Cell Reports Medicine, Volume *5*

Supplemental information

Monocyte bioenergetics: An immunometabolic perspective in metabolic dysfunction-associated steatohepatitis

Moris Sangineto, Martina Ciarnelli, Tommaso Colangelo, Archana Moola, Vidyasagar Naik Bukke, Loren Duda, Rosanna Villani, Antonino Romano, Stefania Giandomenico, Hina Kanwal, and Gaetano Serviddio

Monocyte bioenergetics: an immunometabolic perspective in metabolic dysfunction-associated steatohepatitis

Moris Sangineto[1#*], Martina Ciarnelli[1], Tommaso Colangelo[2,3], Archana Moola[1], Vidyasagar Naik Bukke[1], Loren Duda[4], Rosanna Villani[1], Antonino Romano[1], Stefania Giandomenico[1], Hina Kanwal[1], Gaetano Serviddio[1]

1 C.U.R.E. (University Center for Liver Disease Research and Treatment), Liver Unit, Department of Medical and Surgical Sciences, University of Foggia, Foggia, Italy

2 Department of Medical and Surgical Sciences, University of Foggia, Foggia, Italy

3 Cancer Cell Signalling Unit, Fondazione IRCCS "Casa Sollievo della Sofferenza", San Giovanni Rotondo (FG), Italy

4 Pathology Unit, Department of Clinical and Experimental Medicine, University of Foggia, Foggia, Italy

#Corresponding author: Moris Sangineto, C.U.R.E. (University Center for Liver Disease Research and Treatment), Liver Unit, Department of Medical and Surgical Sciences, University of Foggia, Foggia, Italy

Email: moris.sangineto@unifg.it

***Lead contact**: Moris Sangineto, C.U.R.E. (University Center for Liver Disease Research and Treatment), Liver Unit, Department of Medical and Surgical Sciences, University of Foggia, Foggia, Italy

Email: moris.sangineto@unifg.it

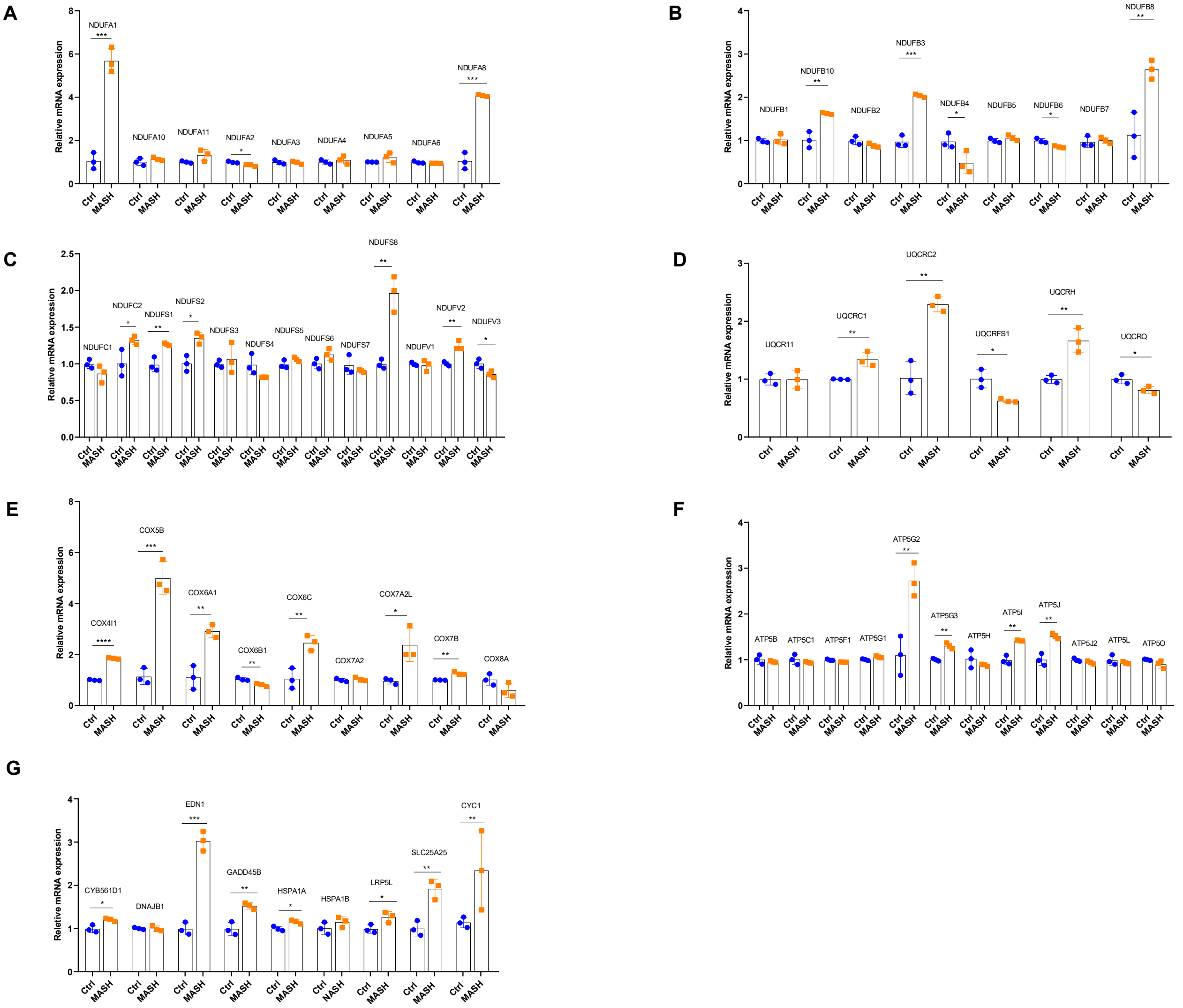

Figure S1. Relative mRNA expression of genes involved in mitochondrial energy metabolism ctrl and MASH Mo. Related to Figure 4.

Determined by qPCR (n= 3 per group) using PrimePCRTM array "Mitochondria Energy Metabolism Plus" (Bio-Rad Laboratories Inc).

Data are expressed in mean ± SEM; *p<0.05; **p<0.01; ***p<0.001, ****p<0.0001 according to two-tailed Stuent's T test.

Genes are listed in supplementary table 2.

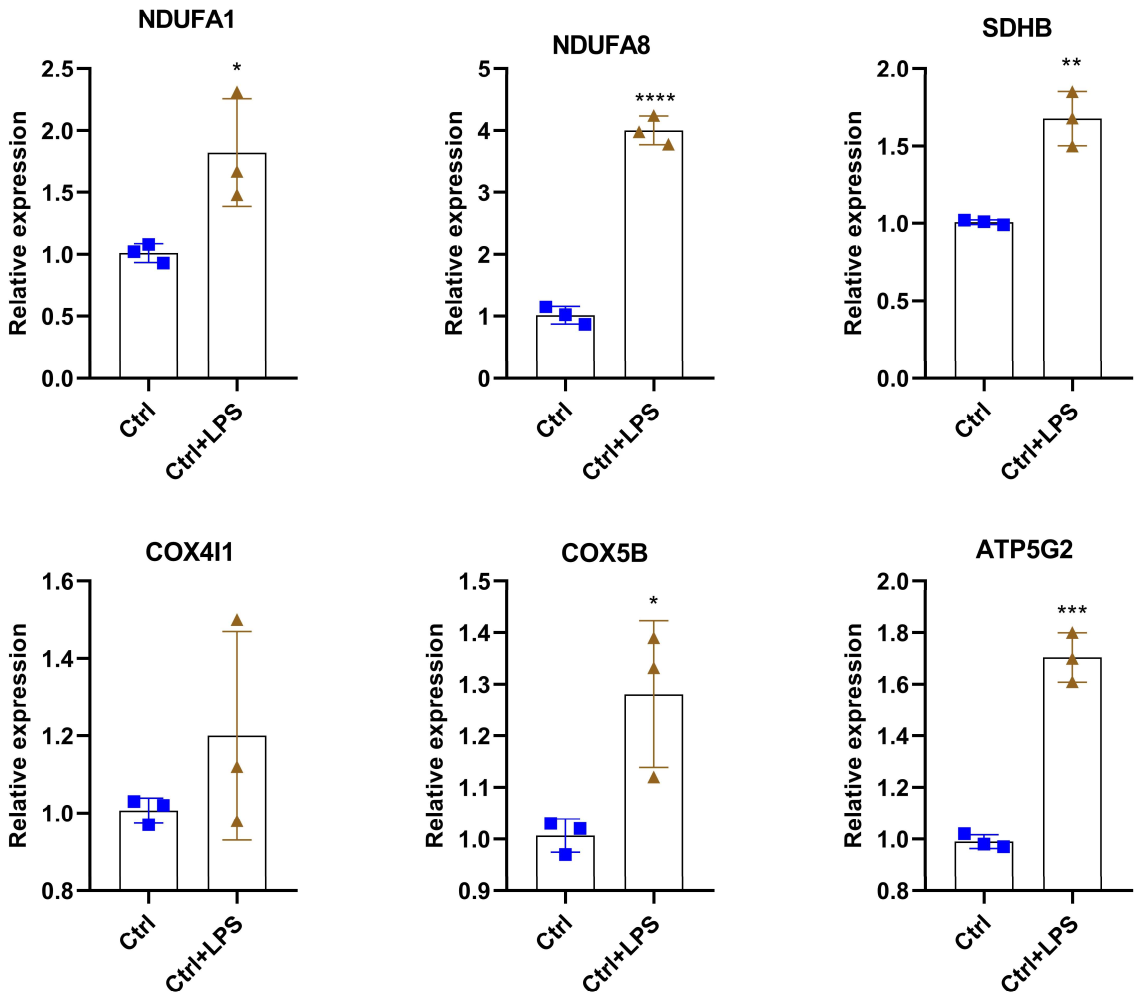

Figure S2. LPS induces expression of ETC subunits in healthy monocytes. **Related to Figure 2 and Figure 4.** Determined by qPCR (n=3 per group).

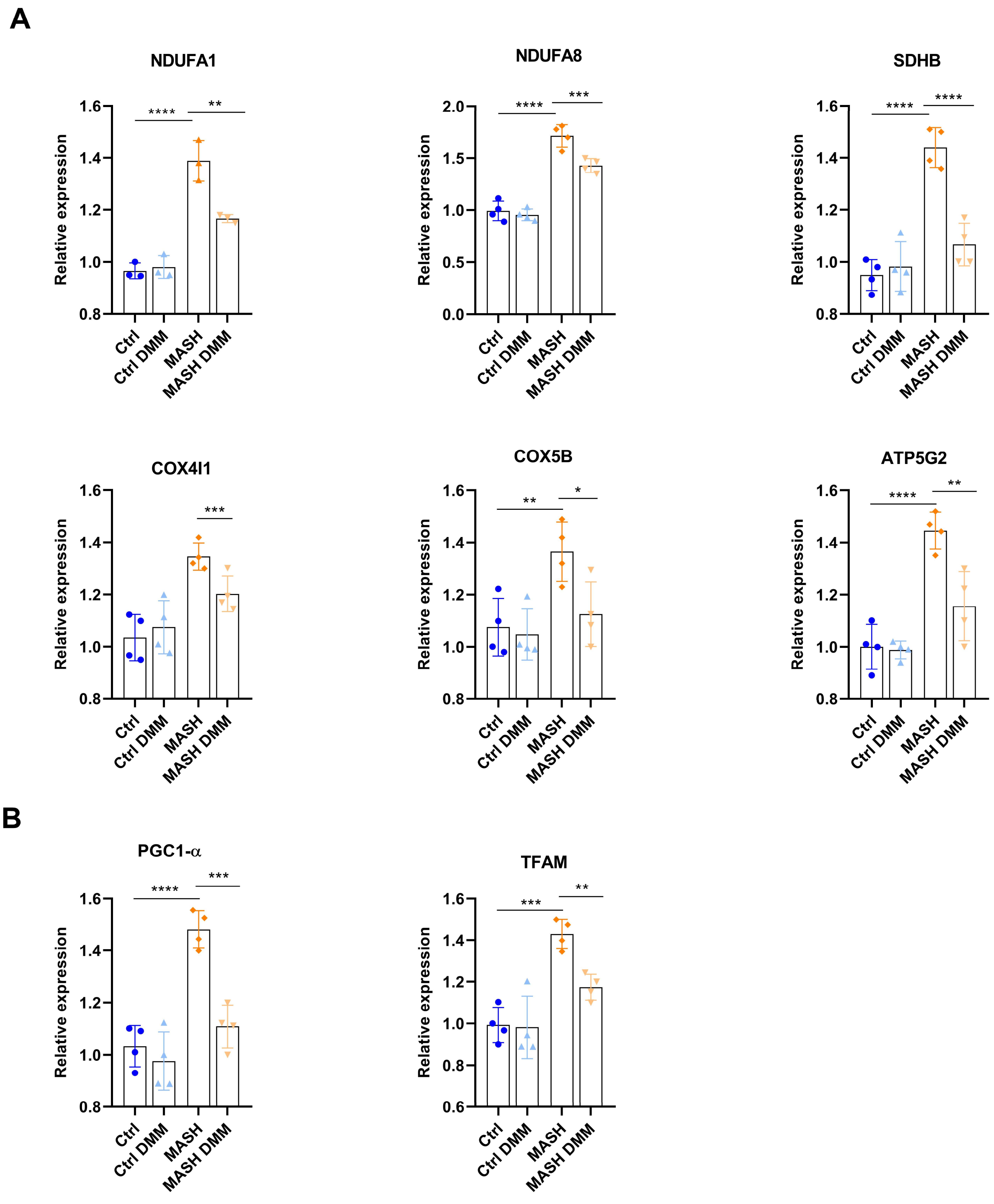

Figure S3. DMM dampens the expression of ETC subunits in MASH Mo. Related to Figure 5.

(A) Relative mRNA expression of ETC subunits ctrl and MASH Mo +/- DMM (10 mM) for 4 h (n= 4 per group), determined by qPCR.

(B) Relative mRNA expression of mitochondrial biogenesis markers (*Pgc1-α* and *Tfam*) in ctrl and MASH Mo +/- DMM (10 mM) for 4 h (n= 4 per group), determined by qPCR.

Data are expressed in mean ± SEM; *p<0.05; **p<0.01; ***p<0.001, ****p<0.0001 according to one-Way ANOVA followed by post hoc analysis (Bonferroni test). DMM, dimethyl malonate; Mo, monocytes; ETC, electron transport chain; PGC-1α, Peroxisome-proliferator-activated receptor-gamma coactivator-1α; TFAM, Transcription factor A, mitochondrial.

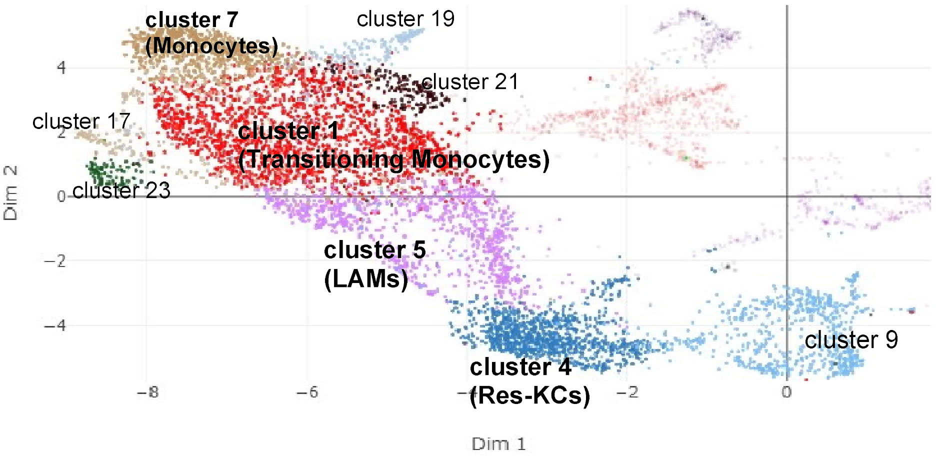

Figure S4. The monocyte-macrophage UMAP based on expression of *Mafb*, *Ly6c2*, *Fcgr1* and *Adgre1.* Related to Figure 6.

UMAP was generated from PCA and data processing of public scRNA-seq dataset (GSE156057) using 24-weeks western diet fed mouse.

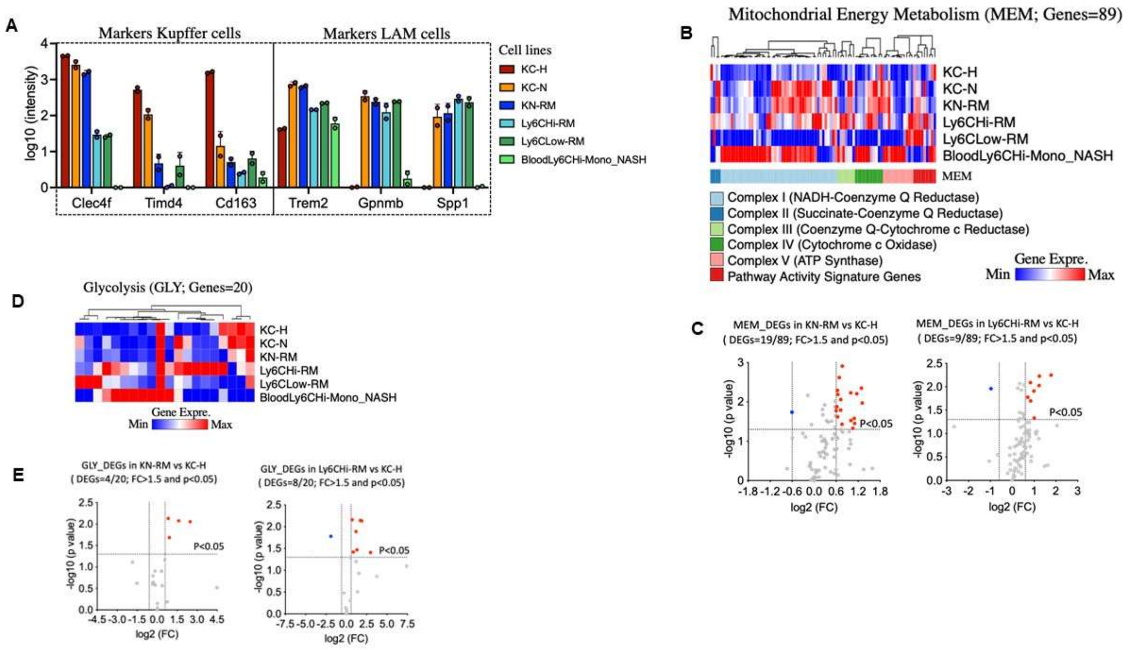

Figure S5. Higher expression of energy pathways in monocyte-derived macrophages compared to normal Kupffer cells in MASH. Related to Figure 6.

(A) Expression of Kupffer cell markers (*Clec4f*, *Timd4* and *Cd163*) and LAM markers (*Spp1, Gpnmb* and *Trem2*) in macrophagic populations isolated from murine MASH livers. (B) Hierarchical clustering and heatmap analyses of 89

genes included in Mitochondrial Energy Metabolism (MEM) pathway. (C) Volcano plot representing MEM_DEGs in KN-RM vs KC-H and $Ly6c^{high}$ vs KC-H with a p value<0.05 and FC>1.5. (D) Hierarchical clustering and heatmap analyses of 20 genes included in Glycolysis (GLY) pathway. (E) Volcano plot representing GLY_DEGs in KN-RM vs KC-H and $Ly6c^{high}$ vs KC-H with a p value<0.05 and FC>1.5. Normalized RNAseq data for different myeloid cell populations in NASH were used from GEO database (GSE128337). Differences were detected with two-tailed Student's T test.

Seidman JS et al[1] firstly identified that KC of healthy livers (KC-H) are substituted during MASH by a macrophagic diversity including KC-NASH (KC-N), and different Mo-MØ: Kupffer-niche recruited macrophages (KN-RM), $Ly6C^{high}$ and $Ly6c^{low}$ recruited macrophages (RM). KC-N are TIM4+ cells, derived from KC progenitors and with transcriptional characteristics near to normal KC-H. KN-RM are TIM4- negative cells, derived from Mo, though transcriptionally more similar to KC-N. KN-RM constitute the 37% of macrophages in MASH model, but their number increases during weeks, occupying a niche within liver sinusoids similar to KC-H and KC-N niche. On the contrary, the $Ly6C^{high/low}$ RM are transcriptionally divergent from KN-RM and occupy niche around large vessels. In particular, $Ly6c^{high}$ RM express several genes common with circulating Mo ($Ly6C^{high}$ blood)[1]. Here, we analysed the public repository of RNA-seq data deposited by Seidman JS et al in order to assess differences in terms of energy metabolism pathways. As expected, Mo-MØ expressed lower or no KC markers (i.e. *Clec-4f*, *Timd4* and *Cd163*) (Supplementary Figure 5A). However, all NASH macrophages, KC-N included, expressed LAM markers (i.e., *Trem2, Gpnmb* and *Spp1*) (Supplementary Figure 5A), highlighting that some contamination existed in the flow cytometry method. Analysing the mitochondrial energy metabolism (MEM) pathway we found that MASH macrophages, and especially Mo-MØ presented an upregulated profile compared to normal KC-H, and very similar to the circulating Mo (Supplementary Figure 5B). Of interest, KC-N, which constitute the TIM4+ cells during MASH, present a profile intermediate between KC-H and KN-RM (Supplementary Figure 5B). While, for unknown reason only the $Ly6C^{low}$ RM showed a strong downregulation of MEM pathway (Supplementary Figure 5B). However, $Ly6C^{low}$ RM represent only the 6% of whole macrophages, with a potential role in tissue repairing mechanisms, while other authors attributed these cells to patrolling monocytes. The volcano plots, showing MEM DEGs of the two most representative Mo-MØ populations (KN-RM vs KC-H, and $Ly6c^{high}$ RM vs KC-H) highlighted a prominent upregulated profile in comparison with normal KC-H. Out of 89 genes composing the MEM pathway, KN-RM had 19 DEGs (18 upregulated and 1 downregulated), while $Ly6c^{high}$ RM had 9 DEGs (8 upregulated and 1 downregulated (Supplementary Figure 5C). In accordance with this, the glycolytic pathway was prominently enhanced in MASH macrophages (Supplementary Figure 5d). As shown by volcano-plot 4 genes out of 20 were significantly up-regulated in KN-RM and 7 genes in $Ly6C^{high}$ RM compared to KC-H (Supplementary Figure 5E). Collectively these results underline the enhancement of MEM and glycolysis pathways in recruited macrophages compared to normal resident macrophages during MASH.

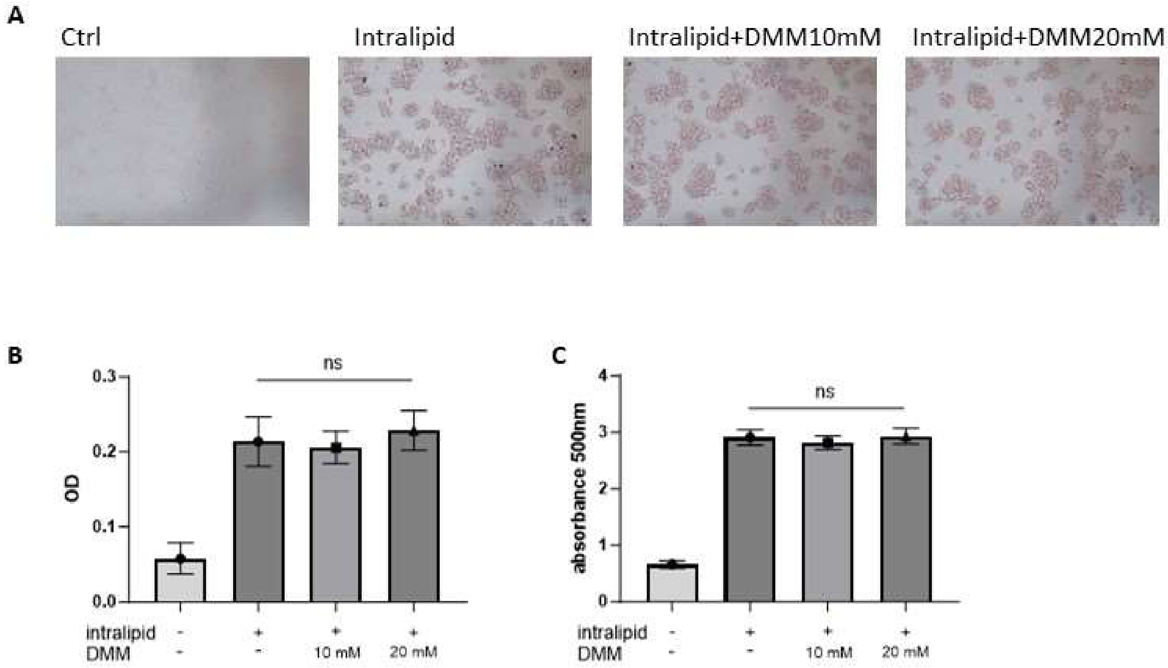

Figure S6. **DMM does not affect hepatocyte lipid accumulation. Related to Figure 7.**

HepG2 cells were exposed to 8% intralipid (Baxter) to induce the steatotic condition and simultaneously treated with DMM (10 mM and 20 mM) for 48 h. A. Pictures of HepG2 stained with Oil Red O to visualize lipid droplets. B. Quantification of optical density of A by FiJi (ImageJ) software. C. Lipid quantification by measurement of Oil Red O absorbance at 500nm.

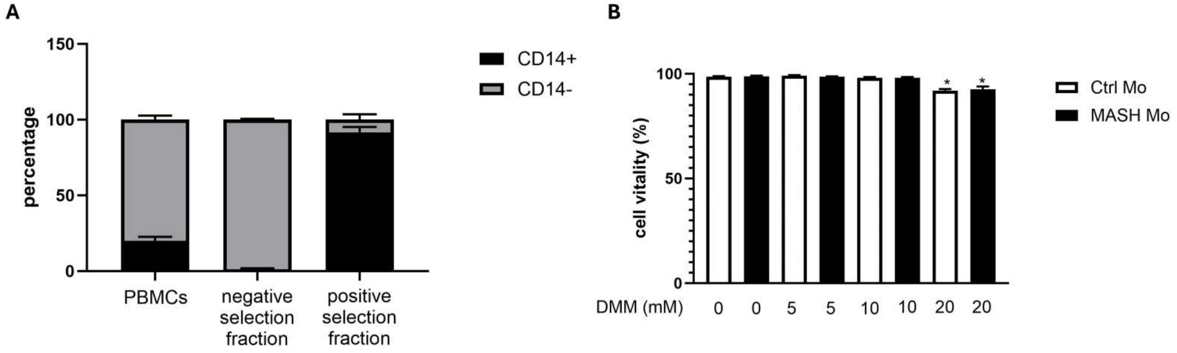

Figure S7. (A) Percentage of CD14+ cells in total PBMCs and fractions obtained after monocyte isolation with EasySep™ Human CD14 Positive Selection Kit II (Stemcell Technologies, Grenoble, France). **Related to Figure 1, Figure 2, Figure 3, Figure 4 and Figure 5.** Determined by FACS analysis on three different isolations.

(B) Cell vitality of ctrl Mo and MASH Mo after 4 hours exposure to increasing concentrations of DMM (dimethyl malonate). **Related to Figure 5.**

Cell vitality was determined with trypan blue staining and living cell count with TC10™ Automated Cell Counter (Bio Rad Laboratories Inc, Segrate (MI), Italy).

Full name	Gene ID
Arrestin domain containing 3	ARRDC3
Ankyrin repeat and SOCS box containing 1	ASB1
ATP synthase, H+ transporting, mitochondrial F1 complex, alpha subunit 1, cardiac muscle	ATP5A1
ATP synthase, H+ transporting, mitochondrial F1 complex, beta polypeptide	ATP5B
ATP synthase, H+ transporting, mitochondrial F1 complex, gamma polypeptide	ATP5C1
ATP synthase, H+ transporting, mitochondrial Fo complex, subunit B1	ATP5F1
ATP synthase, H+ transporting, mitochondrial Fo complex, subunit C1 (subunit 9)	ATP5G1
ATP synthase, H+ transporting, mitochondrial Fo complex, subunit C2 (subunit 9)	ATP5G2
ATP synthase, H+ transporting, mitochondrial Fo complex, subunit C3 (subunit 9)	ATP5G3
ATP synthase, H+ transporting, mitochondrial Fo complex, subunit d	ATP5H
ATP synthase, H+ transporting, mitochondrial Fo complex, subunit E	ATP5I
ATP synthase, H+ transporting, mitochondrial Fo complex, subunit F6	ATP5J
ATP synthase, H+ transporting, mitochondrial Fo complex, subunit F2	ATP5J2
ATP synthase, H+ transporting, mitochondrial Fo complex, subunit G	ATP5L
ATP synthase, H+ transporting, mitochondrial F1 complex, O subunit	ATP5O
Cytochrome c oxidase subunit IV isoform 1	COX4I1
Cytochrome c oxidase subunit Va	COX5A
Cytochrome c oxidase subunit Vb	COX5B
Cytochrome c oxidase subunit VIa polypeptide 1	COX6A1
Cytochrome c oxidase subunit VIa polypeptide 2	COX6A2
Cytochrome c oxidase subunit VIb polypeptide 1 (ubiquitous)	COX6B1
Cytochrome c oxidase subunit Vic	COX6C
Cytochrome c oxidase subunit VIIa polypeptide 2 (liver)	COX7A2
Cytochrome c oxidase subunit VIIa polypeptide 2 like	COX7A2L
Cytochrome c oxidase subunit VIIb	COX7B
Cytochrome c oxidase subunit VIIIA (ubiquitous)	COX8A
Cytochrome b-561 domain containing 1	CYB561D1
Cytochrome c-1	CYC1
DnaJ (Hsp40) homolog, subfamily B, member 1	DNAJB1
Endothelin 1	EDN1
Growth arrest and DNA-damage-inducible, beta	GADD45B
Heat shock 70kDa protein 1A	HSPA1A
Heat shock 70kDa protein 1B	HSPA1B
Low density lipoprotein receptor-related protein 5-like	LRP5L
NADH dehydrogenase (ubiquinone) 1 alpha subcomplex, 1, 7.5kDa	NDUFA1
NADH dehydrogenase (ubiquinone) 1 alpha subcomplex, 10, 42kDa	NDUFA10
NADH dehydrogenase (ubiquinone) 1 alpha subcomplex, 11, 14.7kDa	NDUFA11

NADH dehydrogenase (ubiquinone) 1 alpha subcomplex, 2, 8kDa	NDUFA2
NADH dehydrogenase (ubiquinone) 1 alpha subcomplex, 3, 9kDa	NDUFA3
NADH dehydrogenase (ubiquinone) 1 alpha subcomplex, 4, 9kDa	NDUFA4
NADH dehydrogenase (ubiquinone) 1 alpha subcomplex, 5, 13kDa	NDUFA5
NADH dehydrogenase (ubiquinone) 1 alpha subcomplex, 6, 14kDa	NDUFA6
NADH dehydrogenase (ubiquinone) 1 alpha subcomplex, 8, 19kDa	NDUFA8
NADH dehydrogenase (ubiquinone) 1, alpha/beta subcomplex, 1, 8kDa	NDUFAB1
NADH dehydrogenase (ubiquinone) 1 beta subcomplex, 10, 22kDa	NDUFB10
NADH dehydrogenase (ubiquinone) 1 beta subcomplex, 2, 8kDa	NDUFB2
NADH dehydrogenase (ubiquinone) 1 beta subcomplex, 3, 12kDa	NDUFB3
NADH dehydrogenase (ubiquinone) 1 beta subcomplex, 4, 15kDa	NDUFB4
NADH dehydrogenase (ubiquinone) 1 beta subcomplex, 5, 16kDa	NDUFB5
NADH dehydrogenase (ubiquinone) 1 beta subcomplex, 6, 17kDa	NDUFB6
NADH dehydrogenase (ubiquinone) 1 beta subcomplex, 7, 18kDa	NDUFB7
NADH dehydrogenase (ubiquinone) 1 beta subcomplex, 8, 19kDa	NDUFB8
NADH dehydrogenase (ubiquinone) 1 beta subcomplex, 9, 22kDa	NDUFB9
NADH dehydrogenase (ubiquinone) 1, subcomplex unknown, 1, 6kDa	NDUFC1
NADH dehydrogenase (ubiquinone) 1, subcomplex unknown, 2, 14.5kDa	NDUFC2
NADH dehydrogenase (ubiquinone) Fe-S protein 1, 75kDa (NADH-coenzyme Q reductase)	NDUFS1
NADH dehydrogenase (ubiquinone) Fe-S protein 2, 49kDa (NADH-coenzyme Q reductase)	NDUFS2
NADH dehydrogenase (ubiquinone) Fe-S protein 3, 30kDa (NADH-coenzyme Q reductase)	NDUFS3
NADH dehydrogenase (ubiquinone) Fe-S protein 4, 18kDa (NADH-coenzyme Q reductase)	NDUFS4
NADH dehydrogenase (ubiquinone) Fe-S protein 5, 15kDa (NADH-coenzyme Q reductase)	NDUFS5
NADH dehydrogenase (ubiquinone) Fe-S protein 6, 13kDa (NADH-coenzyme Q reductase)	NDUFS6
NADH dehydrogenase (ubiquinone) Fe-S protein 7, 20kDa (NADH-coenzyme Q reductase)	NDUFS7
NADH dehydrogenase (ubiquinone) Fe-S protein 8, 23kDa (NADH-coenzyme Q reductase)	NDUFS8
NADH dehydrogenase (ubiquinone) flavoprotein 1, 51kDa	NDUFV1
NADH dehydrogenase (ubiquinone) flavoprotein 2, 24kDa	NDUFV2
NADH dehydrogenase (ubiquinone) flavoprotein 3, 10kDa	NDUFV3
Pyrophosphatase (inorganic) 1	PPA1
Succinate dehydrogenase complex, subunit A, flavoprotein (Fp)	SDHA
Succinate dehydrogenase complex, subunit B, iron sulfur (Ip)	SDHB
Succinate dehydrogenase complex, subunit C, integral membrane protein, 15kDa	SDHC
Succinate dehydrogenase complex, subunit D, integral membrane protein	SDHD
Solute carrier family 25 (mitochondrial carrier phosphate carrier), member 25	SLC25A25
Ubiquinol-cytochrome c reductase, complex III subunit XI	UQCR11
Ubiquinol-cytochrome c reductase core protein I	UQCRC1
Ubiquinol-cytochrome c reductase core protein II	UQCRC2
Ubiquinol-cytochrome c reductase, Rieske iron-sulfur polypeptide 1	UQCRFS1
Ubiquinol-cytochrome c reductase hinge protein	UQCRH
Ubiquinol-cytochrome c reductase, complex III subunit VII, 9.5kDa	UQCRQ

Table S1. List of genes from PrimePCR^{TM} array "Mitochondria Energy Metabolism Plus" (Bio-Rad Laboratories Inc, Segrate (MI), Italy). Related to Figure 4.

**Variable**	**N (%)**
**Male**	16 (61.5)
**Hypertension**	10 (38.5)
**Diabetes Mellitus**	20 (77)
**Dyslipidaemia**	12 (45)
	**Mean ± SD**
**Age**	60.24 ± 9.78
**BMI**	30.89 ± 5.34
**AST (U/L)**	51.76 ± 21.32
**ALT (U/L)**	60.20 ± 26.99
**γ-GT (U/L)**	108.25 ± 200.63
**Alkaline phosphatase (U/L)**	97.12 ± 45.22
**Total cholesterol (mg/dL)**	164.25 ± 39.43
**HDL (mg/dL)**	41.32 ± 13.15
**LDL (mg/dL)**	99.25 ± 23.99
**Triglycerides (mg/dL)**	135.24 ± 57.27
**Glucose (mg/dL)**	127.66 ± 59.72
**HbA1c (%)**	6.23 ± 1.21
**Haemoglobin (g/dL)**	13.01 ± 2.51
**Leukocytes (x10$_3$/uL)**	5.42 ± 2.48
**Lymphocytes (%)**	28.95 ± 9.12
**Monocytes (%)**	7.54 ± 2.34
**Platelets (x10$_3$/uL)**	135.95 ± 86.18

Table S2. Baseline characteristics of patients. Related to Figure 1, Figure 2, Figure 3 and Figure 4.

S.No.	Gene	Forward primer	Reverse primer	Source
**1**	Human β-actin	GGCATCGTGATGGACTCC	GCTGGAAGGTGGACAG CGA	Invitrogen
**2**	Human IL-1β	GGCTGCTCTGGGATTCTCTT	TCGTGCACATAAGCCTC GTT	Invitrogen
**3**	Human TNF-α	GTCCTCTTCAAGGGCCAAGG	CTCACAGGGCAATGAT CCCA	Invitrogen
**4**	Human PGC-1α	TGCATGAGTGTGTGCTCTGT	CAGCACACTCGATGTC ACTC	Invitrogen
**5**	Human TFAM	TGATTCACCGCAGGAAAAGC	CGAGTTTCGTCCTCTTT AGCA	Invitrogen
**6**	Mouse β-actin	TATAAAACCCGGCGGCGCA	TCATCCATGGCGAACTG GTG	Invitrogen
**7**	Mouse IL-1β	TGCCACCTTTTGACAGTGATG	TGATGTGCTGCTGCGAG ATT	Invitrogen
**8**	Mouse TNF-α	ACTGAACTTCGGGGTGATCG	CCACTTGGTGGTTTGTG AGTG	Invitrogen
**10**	Mouse CD-163	GGTGCTGGATCTCCTGGTTG	CAGGAGCGTTAGTGAC AGCA	Invitrogen
**11**	Mouse MCP1	CACTCACCTGCTGCTACTCA	GCTTGGTGACAAAAAC TACAGC	Invitrogen
**12**	Human NDUFA1	predesigned	predesigned	Sigma-Aldrich
**13**	Human NDUFA8	Predesigned	Predesigned	Sigma-Aldrich
**14**	Human SDHB	Predesigned	Predesigned	Sigma-Aldrich
**15**	Human COX4I1	Predesigned	Predesigned	Sigma-Aldrich
**16**	Human COX5B	Predesigned	Predesigned	Sigma-Aldrich
**17**	Human ATP5G2	Predesigned	Predesigned	Sigma-Aldrich
**18**	Mouse CCR2	Predesigned	Predesigned	Bio-Rad Laboratories
**19**	Mouse CX3CR1	Predesigned	Predesigned	Bio-Rad Laboratories
**20**	Mouse GPNMB	Predesigned	Predesigned	Bio-Rad Laboratories
**21**	Mouse CLEC4F	Predesigned	Predesigned	Bio-Rad Laboratories

S.No.	Gene	Forward primer	Reverse primer	Source
1	Human β-actin	GGCATCGTGATGGACTCC	GCTGGAAGGTGGACAGCGA	Invitrogen
2	Human IL-1β	GGCTGCTCTGGGATTCTCTT	TCGTGCACATAAGCCTCGTT	Invitrogen
3	Human TNF-α	GTCCTCTTCAAGGGCCAAGG	CTCACAGGGCAATGATCCCA	Invitrogen
4	Human PGC-1α	TGCATGAGTGTGTGCTCTGT	CAGCACACTCGATGTCACTC	Invitrogen
5	Human TFAM	TGATTCACCGCAGGAAAAGC	CGAGTTTCGTCCTCTTTAGCA	Invitrogen
6	Mouse β-actin	TATAAAACCCGGCGGCGCA	TCATCCATGGCGAACTGGTG	Invitrogen
7	Mouse IL-1β	TGCCACCTTTTGACAGTGATG	TGATGTGCTGCTGCGAGATT	Invitrogen
8	Mouse TNF-α	ACTGAACTTCGGGGTGATCG	CCACTTGGTGGTTTGTGAGTG	Invitrogen
22	Mouse TIMD4	Predesigned	Predesigned	Bio-Rad Laboratories
23	Mouse TREM2	Predesigned	Predesigned	Bio-Rad Laboratories
24	Mouse SPP1	predesigned	Predesigned	Bio-Rad Laboratories

Table S3. Primer sequences used in this study. Related to Figure 1, Figure 2, Figure 4, Figure 5 and Figure 7.

Reference List

1. Seidman JS, Troutman TD, Sakai M, Gola A, Spann NJ, Bennett H, Bruni CM, Ouyang Z, Li RZ, Sun X, et al. Niche-Specific Reprogramming of Epigenetic Landscapes Drives Myeloid Cell Diversity in Nonalcoholic Steatohepatitis. Immunity 2020;52:1057-1074 e7.10.1016/j.immuni.2020.04.001
